# Mechanism of Increased Retention of Atomic Hydrogen on Moderately Sulfidated Zero-Valent Iron Surfaces

**DOI:** 10.1021/acs.langmuir.5c04136

**Published:** 2025-10-09

**Authors:** Miroslav Brumovský, Daniel Tunega

**Affiliations:** † Regional Centre of Advanced Technologies and Materials, Czech Advanced Technology and Research Institute (CATRIN), 48207 Palacký University Olomouc, Šlechtitelů 27, 779 00 Olomouc, Czech Republic; ‡ 27270 University of Natural Resources and Life Sciences, Vienna, Department of Ecosystem Management, Climate and Biodiversity, Institute of Soil Research, Peter-Jordan-Straße 82, 1190 Vienna, Austria

## Abstract

Sulfidation represents a promising approach to increase the reactivity, selectivity, and longevity of zero-valent iron (ZVI) in groundwater remediation applications. Recent studies suggest that reductive reactions mediated via adsorbed H* may dominate the degradation of prominent contaminants, such as chlorinated ethenes, on sulfidated ZVI (S-ZVI) surfaces with moderate S coverage, challenging the initially proposed major role of direct electron transfer. This study employs density functional theory to investigate how S coverage and surface corrosion influence H* formation, stability, mobility, and recombination at S-ZVI surfaces at atomic resolution. Our calculations reveal that sulfidation suppresses water adsorption and H* formation via water dissociation, while also weakening H* adsorption affinity on ZVI. However, as surface oxidation also hinders H* adsorption and promotes H* recombination, S-ZVI with moderate (∼ 14 monolayer) S coverage retains more reduced Fe sites, which are favorable for H* adsorption, compared to the corroded ZVI surface. Adsorbed H* at the reduced Fe sites exhibits restricted mobility near S atoms, limiting H* recombination and increasing its availability for contaminant degradation. These findings provide a fundamental mechanistic understanding of increased H* retention at S-ZVI surfaces with moderate S coverage, with implications for the role of H*-mediated reactions in these systems.

## Graphical abstract

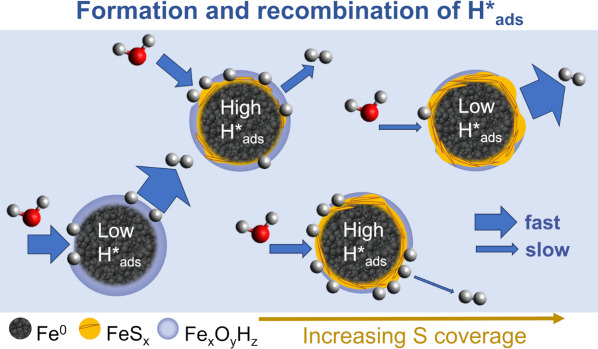

## 1 Introduction

Zero-valent iron (ZVI), including its nanoscale formulations, has been widely used for the in situ reduction of organic and inorganic contaminants in groundwater.^1–3^ However, the performance of this technology in the field has been limited by the rapid reaction of ZVI with water. This reaction leads to particle surface passivation by iron (oxyhydr)­oxides and depletion of their reducing capacity, resulting in reduced contaminant removal efficiency and short reactive lifetime.^4,5^ To overcome these challenges, sulfidation has emerged as a highly promising strategy.^6–8^ Sulfidation enhances the hydrophobicity of the ZVI surface, thereby mitigating water adsorption and its dissociation, i.e., hydrogen evolution reaction (HER), leading to extended operational longevity of ZVI.^9–11^ Furthermore, sulfidated ZVI (S-ZVI) exhibits improved electron selectivity toward contaminants and is more resistant to surface passivation at alkaline pH than pristine ZVI, significantly broadening its application potential in environmental remediation efforts.^12–14^

To further understand and improve these materials, it is essential to examine the fundamental reduction pathways in the ZVI-based systems. These typically occur through two main mechanisms: direct electron transfer or indirect reduction mediated by adsorbed atomic hydrogen (H*_ads_) originating from HER.^15–18^ Despite extensive investigations of S-ZVI materials in the past decade,^6–8^ the relative importance of these pathways in the degradation of contaminants is still not fully understood. Early research, particularly focusing on chlorinated ethenes (CEs), predominantly attributed contaminant reduction on the S-ZVI surface to the direct electron transfer pathway (i.e., β-elimination). This conclusion was based on several observations: (i) FeS_*x*_ phases on the S-ZVI surface better conduct electrons compared to the thicker, less conducting iron (oxyhydr)­oxide passivation layer found on pristine ZVI in aqueous environments;^10,19,20^ (ii) sulfidation promotes the accumulation of acetylene as a dechlorination product as it undergoes only slow hydrogenation;^9,20–22^ and (iii) FeS_*x*_ phases on the S-ZVI surface hinder the adsorption of H*,^10,19,23,24^ implying its lower availability for hydrogenation and hydrogenolysis reactions. Moreover, several studies reported the inhibition of H*-mediated reactions, such as acetylene hydrogenation and chloramphenicol denitration, by ZVI sulfidation.^9,20,21,25–27^

Interestingly, recent works suggested an important role of indirect reduction via H*_ads_ in CE degradation by S-ZVI materials. These studies revealed that the enhancements in CE dechlorination reactivity induced by sulfidation did not correlate with CE reduction potentials (*E* ^0^) or the energies of the lowest unoccupied molecular orbital (*E*_LUMO_), as would be expected for an electron transfer-controlled process.^28–31^ Other investigations have suggested that indirect reduction by H*_ads_ is the dominant dechlorination pathway for less-chlorinated CEs at low S surface coverage.^26,29,31^ Supporting this, cyclic voltammetry analyses by Zhou and co-workers indicated that H*_ads_ accumulates at the S-ZVI surface with moderate S coverage, while its diffusion into the particles is suppressed.^32^ These studies suggest that the predominant contaminant reduction pathway may be dramatically influenced by S loading, particle architecture, and the specific contaminants involved.

Theoretical studies have proven invaluable for revealing the fundamental steps of reaction mechanisms and understanding the effects of controlled surface manipulation on structural, electronic, and magnetic properties, as well as their interactions with atoms and molecules. Quantum chemical methods have been applied to investigate various phenomena, such as iron corrosion,^33,34^ effects of sulfidation on the hydrogen and water adsorption,^10,19,23,35^ and effects of sulfidation on the dechlorination of CEs.^23,36,37^ A comprehensive mechanistic understanding of how S coverage affects the availability of H*_ads_ at the S-ZVI surface is essential for accurately assessing the significance of H*_ads_-mediated pathways in contaminant removal using S-ZVI.

In this study, we employed the density functional theory (DFT) approach to reveal the behavior of H*_ads_ at S-ZVI surfaces with varying S coverage, including its formation via HER, stability, mobility, and recombination. To account for the surface passivation occurring when S-ZVI particles are exposed to water environments, we also systematically examined the impact of surface oxidation and hydroxylation on the generation and fate of H*_ads_. Our DFT results were interpreted alongside existing experimental data to provide a comprehensive understanding of the mechanisms controlling the retention of H*_ads_ on ZVI surfaces with varying S and O/OH coverage. The presented findings may assist in optimizing the S-ZVI reactivity and selectivity for specific environmental remediation applications.

## 2 Materials and Methods

### 2.1 Methods

The computational approach adopted in this study aligns with methodologies established in our earlier works.^23,36^ In summary, all electronic structure calculations were carried out using the spin-polarized plane-wave DFT, as implemented in the Vienna ab initio Simulation Package (VASP).^38–40^ Core–valence electron interactions were treated with the projector-augmented wave (PAW) framework^41,42^ and the electronic exchange–correlation effects were treated using the generalized gradient approximation (GGA), employing the Perdew–Burke–Ernzerhof (PBE) functional.^43^ A kinetic energy cutoff of 400 eV was set for the plane waves in all calculations. To account for long-range van der Waals interactions, the DFT-D3 correction with Becke–Johnson damping was applied.^44,45^ Brillouin zone sampling was performed using a 2 × 2 × 1 Monkhorst–Pack *k*-point grid^46^ for all surface slab models. The electronic and structural optimizations were considered to be converged when total energy changes fell below 10^–^^6^ eV and atomic forces were reduced to less than 0.01 eV/Å, respectively.

The Hubbard *U* correction was employed for the amakinite (Fe­(OH)_2_) slab calculations to address the localized nature of the Fe 3*d* electrons. A *U*_eff_ value of 4.0 eV was applied to the Fe 3*d* states, consistent with prior studies on iron-bearing minerals.^47,48^

The climbing image nudged elastic band method (CI-NEB)^49^ was used to find the minimum energy paths, including transition states (TSs). The reaction coordinates were partitioned into 5–9 images and optimized until either the default convergence thresholds (total energy difference <10^–^^6^ eV and atomic forces <0.01 eV/Å) were met or changes in the energy barrier were below 0.005 eV in more complex cases. The correct identification of TSs was confirmed by vibrational frequency analysis, in which only one imaginary frequency corresponding to the reaction coordinate was obtained. Rarely, the CI-NEB method failed to identify a correct TS; in these cases, the TS geometry was further refined using the dimer method^50^ with the total energy convergence threshold of <10^–^^7^ eV.

To include solvation effects in water adsorption and dissociation reactions, the implicit solvent model VASPsol was employed on the gas-phase-optimized geometries.^51,52^ Solvation effects were omitted in hydrogen adsorption and recombination simulations as prior analysis indicated negligible effects. Hydrogen adsorption energies were referenced to the total electronic energy of an isolated H_2_ molecule in vacuum.

### 2.2 Modeled Systems

#### 2.2.1 Pristine Fe Surface

To study the formation and fate of H*_ads_ on a reference pristine Fe surface, a slab model of the (110) facet, which is the α-Fe closest-packed surface with the lowest surface energy,^53^ was employed. The Fe(110) slab model consisted of a 4 × 5 supercell with lateral dimensions 15.881 × 13.889 Å with three atomic planes in the direction parallel to the surface. This model has been constructed and validated in our previous studies.^54,55^

#### 2.2.2 Fe Surfaces with Varying S Coverage

To investigate the effect of S coverage on the behavior of H*_ads_, a set of three Fe(110) surfaces doped with several S atoms on the hollow sites (also termed as “long-bridge” (LB) sites^56^) in a regular fashion was generated using the pristine Fe slab model. These three models represented (i) 1/8 monolayer coverage (termed as “S_1/8 ML_-Fe­(110)”), (ii) 1/4 monolayer coverage (termed as “S_1/4 ML_-Fe­(110)”), and (iii) 1/2 monolayer coverage (termed as “S_1/2 ML_-Fe­(110)”). The generated models exemplify low, moderate, and high S coverages and have been developed in our recent studies to showcase the intrinsic effects of sulfidation on the S-ZVI surface reactivity with CEs in electron-transfer-mediated reactions.^23,36^ Assuming the particle BET specific surface area^21^ of ∼30 m^2^ g^–1^ and the deposition of all S atoms in a single atomic layer, these surfaces can serve as representative models of S-ZVI nanoparticles prepared by the postsulfidation method with S/Fe molar ratios of 0.007, 0.014, and 0.028. This range of S/Fe molar ratios corresponds well to the typical S/Fe ratios investigated in experiments, exhibiting slower HER and enhanced performance in contaminant removal.^21^

#### 2.2.3 Fe Surfaces with Varying O/OH Coverage

To compare the effects of surface sulfidation and corrosion, we also constructed models of Fe(110) surfaces doped with O atoms and OH groups in a regular fashion. These models had a topology analogous to the 1/8 and 1/4 monolayer coverage by S atoms, resulting in “O_1/8 ML_-Fe­(110)”, “OH_1/8 ML_-Fe­(110)”, “O_1/4 ML_-Fe­(110)”, and “OH_1/4 ML_-Fe­(110)” surface slab models. Our attempts to construct oxidized/hydroxylated surfaces with the same topology as the S_1/2 ML_-Fe­(110) model led to the structural reorganization of O/OH dopants into a hexagonal pattern as they migrated into 3-fold hollow (3FH) sites. Therefore, these surface models were not further considered in this study, as they will not allow for a direct comparison of dopant effects due to altered topology.

#### 2.2.4 Mackinawite (FeS_m_)

A diamagnetic 4 × 4 supercell surface slab model of FeS_m_(001) was used as a representation of 1:1 iron sulfide formed on the surface of ZVI particles upon sulfidation, which has been frequently reported in particles treated in solutions of reduced sulfur species (i.e., postsulfidation).^8^ The topology of the topmost S layer of FeS_m_ is similar to that of the S_1/2 ML_-Fe­(110) model, with the main difference being the presence of an S atom instead of an Fe atom below the hollow adsorption sites. This model has been developed and validated in our previous works.^54,55^

#### 2.2.5 Amakinite (Fe­(OH)_2_)

Amakinite has been identified as the primary product of iron corrosion in water under anaerobic conditions.^33^ Hence, a 4 × 4 supercell surface slab model of this mineral was constructed to serve as a proxy for the freshly corroded ZVI surface. The bulk structure of amakinite^57^ was used for the construction of the slab model.

Structures of all slab models are shown in Figure S1. All surface models contained at least a 25 Å-thick vacuum layer in the direction perpendicular to the surface to decouple adjacent slabs. Models were allowed to fully relax during H* and water adsorption and CI-NEB calculations with constant lattice parameters.

## 3 Results and Discussion

### 3.1 Sulfidation Suppresses Water Adsorption and Dissociation at the ZVI Surface

The abundance of H*_ads_ at the ZVI surface is controlled by the extents of its formation and loss processes.^32^ H*_ads_ is primarily generated via HER, which is an integral part of ZVI corrosion in water environments. Water adsorption at the ZVI and S-ZVI surfaces is the first step toward the formation of H* via HER.^34^ Our DFT results reveal that increasing S coverage on the Fe(110) surface leads to less favorable water adsorption energies (Δ*E*_ads_), increasing from −59.0 kJ mol^–1^ at the pristine Fe(110) surface to −13.7 kJ mol^–1^ at the S_1/2 ML_-Fe­(110) surface (Figure 1A and Table S1) with the inclusion of the implicit solvent effect. At the FeS_m_(001) surface, water Δ*E*_ads_ reached only −8.5 kJ mol^–1^. This is in line with experimental studies reporting an increase in ZVI surface hydrophobicity after sulfidation,^9,10,58^ as well as theoretical calculations revealing weaker water adsorption energies in the proximity of S atoms on the Fe surface.^23,35^ The previously reported water Δ*E*_ads_ values at the pristine Fe(110) surface ranged −36.7 to −51.5 kJ mol^–1^,^23,34,56^ while that at the FeS_m_(001) surface was of −16.4 kJ mol^–1^,^59^ which also aligns with the energies calculated in this study. The slightly different Δ*E*_ads_ values reported here likely stem from the different methodologies used to treat dispersion interactions in prior studies.

**Figure 1:**
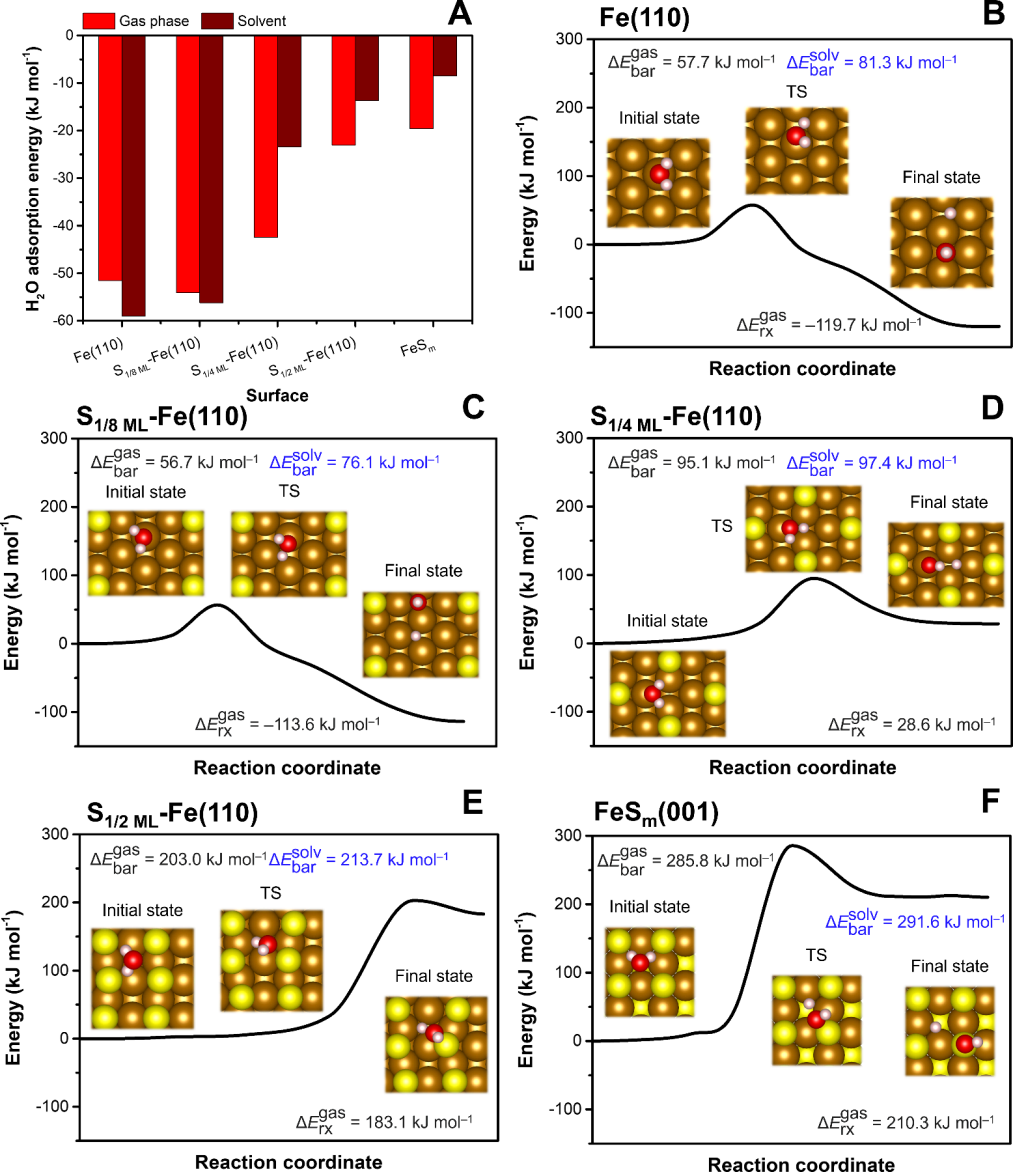
Adsorption and dissociation of water at different surfaces: (A) Adsorption energy of water on the studied surfaces in the gas phase and in solvent (water); Reaction profiles of water dissociation at the (B) pristine Fe(110) surface, (C–E) S-doped Fe(110) surfaces with varying S coverage, and (F) FeS_m_(001) surface. CI-NEB calculations were performed in the gas phase (values in black). The results of the transition state (TS) vibrational frequency analysis are shown in Table S2. The solvent effect on the reaction barrier was included using a continuum solvation model with the structures of reactant and TS taken from the CI-NEB calculation (values in blue).

The preferred H_2_O adsorption configuration varied among different surface models (Figure S2): at the Fe(110), S_1/8 ML_-Fe­(110), and S_1/4 ML_-Fe­(110) surfaces, the most stable H_2_O adsorption configuration was at the top (T) Fe site, with a H_2_O molecule oriented parallel to the Fe surface and the Fe–O_W_ distances between 2.18–2.26 Å. At higher S coverages (i.e., at the S_1/2 ML_-Fe­(110) and FeS_m_(001) surface models), water molecules adsorbed at larger distances (4.16–4.21 Å) with hydrogen atoms oriented toward surface sulfur atoms. The gas-phase water adsorption configurations at the investigated surfaces are in a remarkable agreement with previous DFT studies.^11,23,34,35,56,59^

The adsorbed water molecules can further dissociate into H* and OH^–^ according to the Volmer reaction (eq 1):

$${\mathrm{Fe}}^{0}+H_{2}O+e^{-}\to \mathrm{Fe}-H^{*}+{{\mathrm{OH}}^{-}}_{\left(\mathrm{ads}\right)}$$ 1

The calculated water dissociation barriers (Δ*E*_bar_) at surfaces with varying S coverage followed the same trend observed for water Δ*E*_ads_ (Figure 1B–F). Both water adsorption and dissociation at the S_1/8 ML_-Fe­(110) surface exhibited an energy profile comparable to that at the pristine Fe(110) surface, pointing to only local effects of S atoms on this S-ZVI surface, representing low S coverage. In contrast, the water dissociation Δ*E*_bar_ increased significantly at the S_1/4 ML_-Fe­(110) surface and reached values >200 kJ mol^–1^ at the S_1/2 ML_-Fe­(110) and FeS_m_(001) surfaces, suggesting that water dissociation is inhibited at surfaces with high S coverage. Note that the calculated gas-phase water dissociation Δ*E*_bar_ at the pristine Fe(110) surface (57.7 kJ mol^–1^) was slightly smaller than the previously reported values of 65.6 ^56^ and 76.2 ^34^ kJ mol^–1^, which can be attributed to the different treatment of dispersion interactions and varying product configurations in prior works.

The poorer feasibility of water dissociation at surfaces with increasing S coverage is also evident from the geometry of reaction products and the reaction energy (Δ*E*_rx_) values (Figure 1B–F): While H_2_O dissociation at pristine Fe(110) and S_1/8 ML_-Fe­(110) surfaces is thermodynamically favorable, yielding H and OH chemisorbed in 3FH sites, Δ*E*_rx_ reaches positive values at the S_1/4 ML_-Fe­(110) surface and surfaces with higher S coverage. On these surfaces, the OH group is chemisorbed either at the Fe T site (S_1/4 ML_-Fe­(110) surface) or at an S atom (S_1/2 ML_-Fe­(110) and FeS_m_(001) surfaces).

Overall, these results reaffirm the critical role of sulfidation in decreasing the S-ZVI surface interactions with water and suppressing HER, further indicating that the hydrogen evolution rate at S-ZVI surfaces is smaller compared to nonsulfidated ZVI.

### 3.2 Oxidation of the ZVI Surface Promotes Further Corrosion and H* Formation through Facilitated Water Dissociation

Due to its high reactivity, ZVI rapidly develops a surface passivation layer consisting of iron (oxyhydr)­oxides upon contact with aqueous media.^60^ The minerals and amorphous phases in this layer are hydrophilic, as supported by calculated water adsorption energies at oxidized and hydroxylated Fe(110) surfaces, which were comparable to or more favorable than those at the pristine Fe(110) surface (Table S1 and Figures S3–S5). In contrast to sulfidation, preadsorbed oxygen atoms, hydroxyl groups, and even water molecules have been found to accelerate ZVI surface corrosion by lowering water dissociation barriers.^56,61^

To evaluate the feasibility of water dissociation at moderately corroded ZVI surfaces, we calculated its reaction profiles at the O_1/4 ML_-Fe­(110) and OH_1/4 ML_-Fe­(110) surface slab models. The reaction barriers at these surfaces were lower than those calculated at the pristine Fe(110) surface. At the O_1/4 ML_-Fe­(110) surface, we observed an O-assisted dissociation into OH + OH with a Δ*E*_bar_ value of 51.1 kJ mol^–1^ (Figure 2A). This reaction is in line with the facilitated water dissociation at the O precovered ZVI surface.^56^ While this reaction does not yield H*_ads_ per se, it creates OH sites favorable for water dissociation via H^+^ transfer as observed at the OH_1/4 ML_-Fe­(110) surface (Figure 2B). In such a reaction, a water molecule from solution first interacts with the preadsorbed OH group via a hydrogen bond and then undergoes dissociation coupled with the acceptance of H^+^ from the OH group with a Δ*E*_bar_ value of 47.6 kJ mol^–1^. The net result of this reaction is facilitated production of H*_ads_ and promotion of further ZVI surface corrosion. The analysis of HER feasibility shows that sulfidation has an opposite effect on H*_ads_ formation compared to oxidation. To explain increased H*_ads_ retention at the S-ZVI surface, the fate of H*_ads_ at the sulfidated and corroded ZVI surfaces has to be further considered.

**Figure 2:**
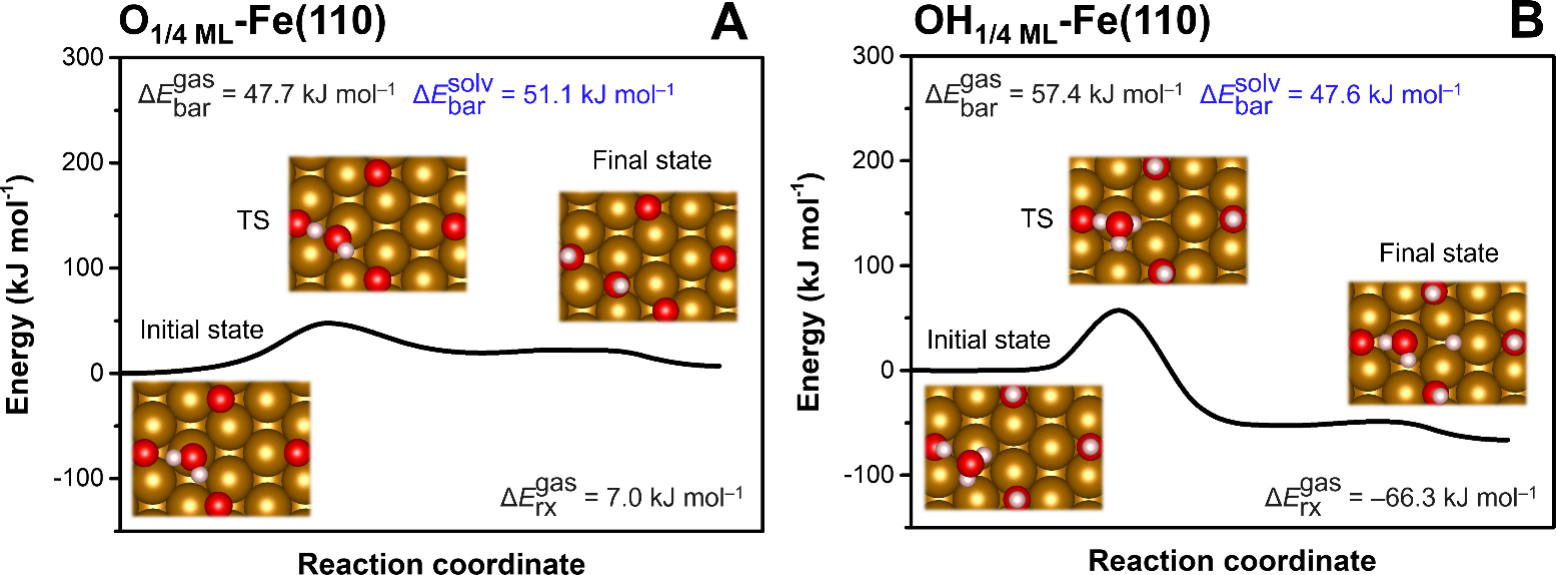
Reaction profiles of water dissociation at moderately corroded ZVI surfaces: (A) O_1/4 ML_-Fe­(110) surface and (B) OH_1/4 ML_-Fe­(110) surface. CI-NEB calculations were performed in the gas phase (values in black). The results of transition state (TS) vibrational frequency analysis are shown in Table S2. The solvent effect on the reaction barrier was included using a continuum solvation model, with the structures of reactant and TS taken from the CI-NEB calculation (values in blue).

While our findings are in perfect agreement with corrosion studies of ZVI and S-ZVI systems, two key simplifications should be acknowledged: (i) ZVI surface corrosion and H* formation typically involve additional reactions beyond the Volmer step and their relative importance depends on pH and the presence of solutes;^4,62–64^ and (ii) the self-promoting effect of corrosion persists only while Fe^0^ remains exposed on the surface. Once the ZVI surface becomes covered by a thick layer of iron (oxyhydr)­oxides, passivation can inhibit further corrosion. Under such conditions, commonly observed at pH > 9, S-ZVI may exhibit faster H* formation.^12^

### 3.3 Low to Moderate S Coverages Retain Fe Sites Favorable for H* Adsorption with Low Migration Barriers

Once formed through the HER, H*_ads_ may undergo different processes at metal surfaces, including surface migration, diffusion into the bulk, and/or recombination to form H_2_. To assess the importance of these processes in the fate of H*_ads_ in S-ZVI systems, we calculated the H*_ads_ adsorption energies at all inequivalent sites of Fe(110) surfaces with varying S coverage, determined migration barriers between distinct surface sites, and established minimum energy paths for the hydrogen recombination reactions (see Section 3.5). Note that H* absorption into bulk was not considered in this study, as this process was found to be negligible in S-ZVI systems.^32^

The Fe(110) surface features four different potential H* binding sites: the top (T), long-bridge (LB), short-bridge (SB), and 3-fold hollow site (3FH) (Figure 3A).^56^ The most stable H*_ads_ adsorption complex was found at the 3FH site with an Δ*E*_ads_ value of −78.8 kJ mol^–1^ (Figure 3B and Table S3). H* adsorption is slightly weaker at the LB and SB sites (−73.3 and −62.2 kJ mol^–1^, respectively), while no stable configuration could be calculated for the T site, as H* migrated to the nearest 3FH site upon structural relaxation. The LB and SB sites can be seen as TSs in the migration of H*_ads_ from one 3FH site to another. Due to small energy differences (Figure 3C), H*_ads_ can migrate easily on the pristine Fe(110) surface. These results are in perfect agreement with previous computational studies.^56,65,66^

**Figure 3:**
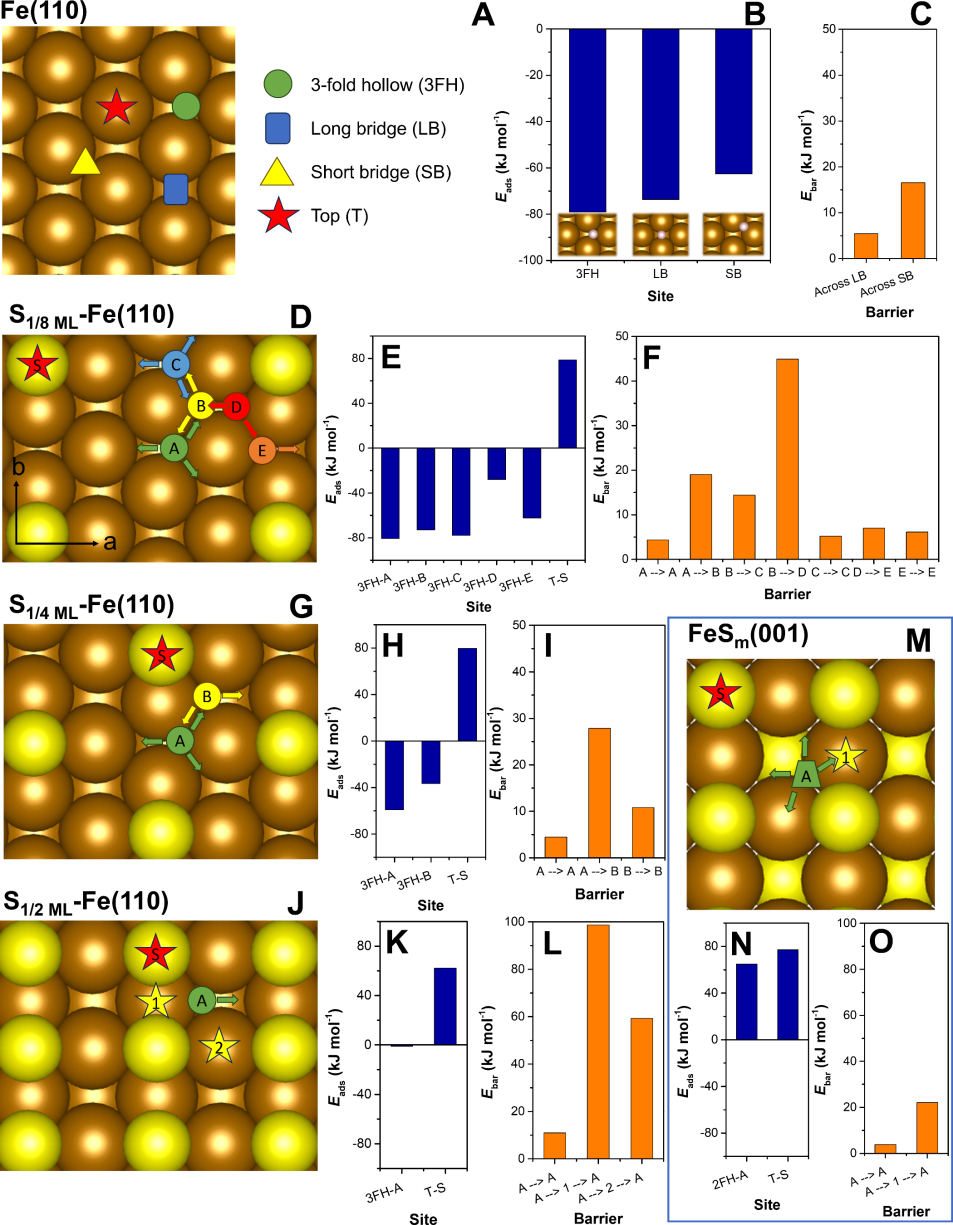
Hydrogen adsorption at pristine and sulfidated Fe-bearing surfaces and sites, including Fe(110), S_1/8 ML_-Fe­(110), S_1/4 ML_-Fe­(110), S_1/2 ML_-Fe­(110), and FeS_m_(001): (A, D, G, J, and M) Possible adsorption configurations of H*_ads_ at varying surfaces, (B, E, H, K, and N) H* adsorption energies at these sites, and (C, F, I, L, and O) migration barriers between H*_ads_ adsorption sites. H* adsorption at the “D” site of S_1/8 ML_-Fe­(110) was determined in a single-point calculation, as it migrated to the nearby “B” site upon structural relaxation. The arrows indicate unrestricted H*_ads_ migration pathways at room temperature (Δ*E*_bar_ < 30 kJ mol^–1^).

Figure 3 and Table S3 display the Δ*E*_ads_ values and migration barriers of H* at Fe(110) surfaces with increasing S coverage and at the FeS_m_(001) surface. Note that only energies of 3FH sites at Fe(110) surfaces are shown, while the energies of LB and SB sites can be derived from the energies of TSs between individual 3FH sites. Due to the reduced symmetry incurred by the presence of S atoms, the TSs do not, however, always correspond to the middle of the reaction coordinate.

The Δ*E*_ads_ values of H* at the most favorable sites of each surface model reveal a consistent trend that sulfidation weakens the H* adsorption affinity to the surface: Δ*E*_ads_ increased in the order of −78.8 to −80.6 < – 59.2 ≪ −1.1 ≪ 64.8 kJ mol^–1^ for the pristine Fe(110), S_1/8 ML_-Fe­(110), S_1/4 ML_-Fe­(110), S_1/2 ML_-Fe­(110), and FeS_m_(001), respectively. This indicates that H* adsorption is thermodynamically favorable only at surfaces with low (S_1/8 ML_-Fe­(110)) to moderate (S_1/4 ML_-Fe­(110)) S coverage.

The S_1/8 ML_-Fe­(110) surface with low S coverage exhibited localized effects, which were significant only near sulfur atoms. In contrast, the Δ*E*_ads_ of H*_ads_ at sites farther from the S atoms were comparable to the 3FH site of the pristine Fe(110). The local effects arise from a combination of steric hindrance (Table S4) and dilution of electron density caused by charge transfer to the more electronegative S atoms (Figure S6). The electron redistribution is associated with a slight downward shift of the *d*-band center relative to the Fermi level (Figure S7), a trend that became more pronounced with higher S coverage. On the moderately sulfidated ZVI surface exemplified here by the S_1/4 ML_-Fe­(110) surface model, the Δ*E*_ads_ values of H*_ads_ were about 25–50% higher compared to the 3FH site of the pristine Fe(110) at all 3FH sites.

At the S_1/2 ML_-Fe­(110) surface model representing a high sulfidation degree, the Δ*E*_ads_ of H*_ads_ was close to zero, suggesting that the H* adsorption at this surface is equally favorable as the presence of H in a gas-phase H_2_ molecule. The most favorable H* adsorption at the FeS_m_(001) surface occurred at a 2-fold hollow (2FH) site between two Fe atoms, with a positive Δ*E*_ads_, indicating that H* adsorption is not thermodynamically favorable. This unfavorable H* adsorption at the FeS_m_(001) surface aligns with the reported reactivity of mackinawite with contaminants, which typically involves the direct electron transfer mechanism.^26,67^

Interestingly, Zhou and co-workers^32^ proposed that H*_ads_ is stabilized on the S-ZVI surface by binding to S atoms, based on an observed peak in the Raman spectrum between 2400 and 2500 cm^–1^ assigned to the S–H stretching vibration. Our results do not align with this conclusion as H* adsorption to S atoms in all studied surface models consistently yielded unfavorable adsorption energies (Figure 3). While solvation can stabilize the S–H bonds, as confirmed in calculations with both implicit and explicit solvation models (Figure S8A,B), our findings suggest that H* adsorption to hollow Fe sites is more favorable. Moreover, H* adsorbed at Fe hollow sites will be more readily involved in reduction/radical reactions due to its negative charge (Figure S8C). In contrast, the S–H bond may favor heterolytic cleavage, yielding H^+^ in a water environment.

The low H*_ads_ migration barriers at Fe(110) surfaces with low to moderate S coverage (Δ*E*_bar_ typically <30 kJ mol^–1^) indicate that H*_ads_ is relatively mobile. There are two requirements for H*_ads_ to migrate easily at the Fe(110) surface: (i) migration proceeds far more rapidly through the LB or SB sites than through top sites, so the former sites must remain accessible; and (ii) H*_ads_ should avoid passing in the immediate vicinity of S atoms. The constraining effect of S atoms is evident at site D at the S_1/8 ML_-Fe­(110) surface, which is much less thermodynamically favorable for H* adsorption than the surrounding 3FH sites. The H*_ads_ migration from site B to this site reaches a Δ*E*_bar_ value of 44.9 kJ mol^–1^ (Figure 3E, F). Given the uneven spacing of S atoms on this surface, H*_ads_ migration is hindered along the *a* direction (horizontal in Figure 3D) but remains largely unaffected along the *b* direction. By contrast, at the S_1/4 ML_-Fe­(110) surface, H*_ads_ migration is expected to occur uniformly in all diagonal directions because of the higher surface symmetry. Interestingly, the barriers for H*_ads_ crossing through the 3FH sites near S atoms (e.g., site B in Figure 3G) are lower than those at the corresponding sites (site D) at the S_1/8 ML_-Fe­(110) surface. This is due to the reduced stabilization of H*_ads_ at the nearby A sites as a result of the larger effect of S atoms.

At the S_1/2 ML_-Fe­(110) surface, H*_ads_ migration is strongly hindered, as all LB and SB sites are blocked by S atoms. As a result, H*_ads_ has to pass through Fe top sites 1 or 2 adjacent to S atoms (Figure 3J) with Δ*E*_bar_ values of 98.7 and 59.3 kJ mol^–1^, respectively. Although a similar trend might be intuitively anticipated for the FeS_m_(001) surface, its intrinsically unfavorable H* adsorption energies lower the H*_ads_ migration barrier to just 22.1 kJ mol^–1^ across Fe top site 1 (Figure 3M), thereby facilitating hydrogen recombination as discussed in subsequent sections. A summary of the nearest distances between S atoms, surface density of H* adsorption sites, their connectivity, and H*_ads_ migration feasibility is shown in Table S5 for each surface model.

### 3.4 Iron Corrosion Decreases H* Adsorption Affinity

While several recent studies have explored the intrinsic effects of S atoms on H* adsorption strength at the ZVI surface,^10,19,23,24^ the influence of surface corrosion on H* adsorption has been largely overlooked. To investigate the impact of surface corrosion on the stability of H*_ads_, we performed analogous calculations to those at the S_1/8 ML_-Fe­(110) and S_1/4 ML_-Fe­(110) surfaces, substituting S atoms with O atoms and OH groups to simulate low and moderately corroded Fe surfaces.

Introducing O/OH to the ZVI surface produces a comparable trend in H*_ads_ Δ*E*_ads_ as sulfidation (Figure 4 and Table S6). At the Fe(110) surface with 1/8 O/OH monolayer coverage, only localized effects of these atoms/groups were detectable, with Δ*E*_ads_ of H*_ads_ ∼ −80 kJ mol^–1^ at the O–H*_ads_ distances >3 Å. However, at the 14 monolayer coverage, the Δ*E*_ads_ values were weakened by half to about −60 kJ mol^–1^. The local effects of the OH/O bond are less noticeable than those of the S atoms due to the smaller atomic size of the O bond and its ability to migrate around the LB sites to adjacent 3FH sites, thereby minimizing steric effects. This distinction is most evident in the feasibility of H*_ads_ migration along the *a* direction at 1/8 monolayer coverage: whereas S atoms partially hinder migration (Figure 3F), O/OH species arranged in the same pattern still allow easy H*_ads_ movement (Figure 4C,F). Nonetheless, the presented data point to low H* adsorption feasibility at extensively corroded ZVI surfaces, which is in perfect agreement with previous studies investigating the effect of oxygen coverage on the retention of H*_ads_ at iron surfaces.^33,56^

**Figure 4:**
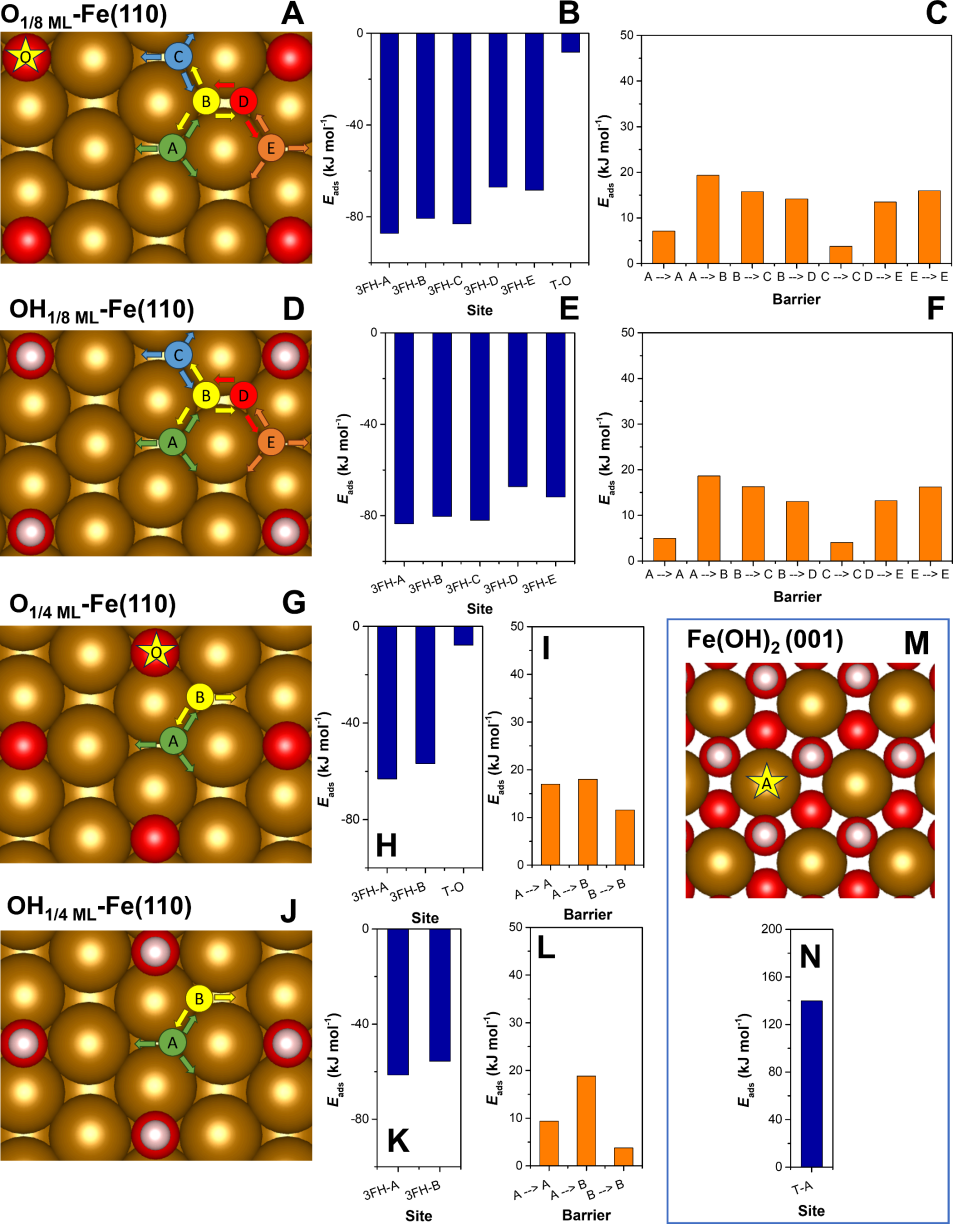
Hydrogen adsorption at oxidated and hydroxylated Fe-bearing surfaces and sites, including O_1/8 ML_-Fe­(110), OH_1/8 ML_-Fe­(110), O_1/4 ML_-Fe­(110), OH_1/4 ML_-Fe­(110), and Fe­(OH)_2_(001): (A, D, G, J, and M) Possible adsorption configurations of H*_ads_ at varying surfaces, (B, E, H, K, and N) H* adsorption energies at these sites, and (C, F, I, and L) migration barriers between H*_ads_ adsorption sites. The arrows indicate unrestricted H*_ads_ migration pathways at room temperature (Δ*E*_bar_ < 30 kJ mol^–1^).

Unlike S atoms, the O sites exhibited favorable H* adsorption. Although the Δ*E*_ads_ values were much higher (∼ −12 kJ mol^–1^) than those for Fe 3FH sites, solvation effects are expected to enhance their stability, as previously demonstrated for S–H bonds (Figure S8). However, the strong polarity of the O–H bonds reduces the ability of H*_ads_ to participate in reductive reactions as H*.

To simulate a highly corroded ZVI surface, we constructed a surface slab model of amakinite (Fe­(OH)_2_), which has been identified as the primary product of iron corrosion in water under anaerobic conditions.^33^ The Δ*E*_ads_ of H*_ads_ on this surface at a top Fe site was 140 kJ mol^–1^, corroborating the unfavorable H*adsorption (Figure 4N). As a result, the TS calculation for H*_ads_ migration between neighboring top Fe sites revealed a tendency for H* to recombine with H* from nearby OH groups, favoring H_2_ desorption over surface migration (Figure S9). A summary of the nearest distances between the O/OH atoms, surface density of H* adsorption sites, their connectivity, and H*_ads_ migration feasibility is shown in Table S7 for each surface model.

To summarize, both sulfidation and corrosion of ZVI decrease the surface affinity for H*_ads_. Since H*_ads_ remains relatively mobile at mildly to moderately sulfidated or corroded surfaces, it is reasonable to propose that during continued corrosion coupled with the HER, the generated H*_ads_ migrates toward more preserved ZVI surface regions. These more pristine areas provide adsorption sites with lower Δ*E*_ads_ values, making them more favorable for H*_ads_ binding.

### 3.5 H* Adsorption Energies Primarily Control H* Recombination on the S-ZVI Surface

The recombination of H*_ads_ to form H_2_ represents the terminal loss process of H*_ads_ (excluding reactions with species other than water). This can proceed through either the Tafel (eq 2) or the Heyrovsky (eq 3) reaction:^32,68,69^

$$2\mathrm{Fe}-H^{*}\to 2\mathrm{Fe}+H_{2}$$ 2

$$\mathrm{Fe}-H^{*}+H_{2}O+e^{-}\to \mathrm{Fe}+H_{2}+{\mathrm{OH}}^{-}$$ 3

To evaluate the feasibility of hydrogen recombination at pristine and sulfidated ZVI, we computed the Tafel reaction profiles with the initial configuration having two H*_ads_ adsorbed at opposite 3FH sites of a single Fe atom. This geometry corresponds to the product of spontaneous H_2_ dissociation observed at the Fe(110) and S_1/8 ML_-Fe­(110) surfaces. While the Heyrovsky reaction was not explicitly calculated, it is reasonable to expect a similar trend in activation barriers, as surface S atoms repel both H* and water.

As expected, the Tafel recombination reactions exhibited varying feasibilities and energy barriers across different surface models, depending on the S surface coverage. At the Fe(110) and the S_1/8 ML_-Fe­(110) surfaces, neither the CI-NEB nor the dimer method was able to identify a TS geometry with negative curvature and an imaginary frequency along the H*_ads_ recombination reaction coordinate (Figure 5A, B, and Table S8), but the reaction was thermodynamically unfavorable, with a positive energy of 154 kJ mol^–1^. These reactions effectively represent the opposite of spontaneous H_2_ dissociation, which was observed during the structural optimization of adsorbed H_2_ at these surfaces. In these cases, the energy barrier for H_2_ recombination corresponds to the sum of the Δ*E*_ads_ values of the two H*_ads_, slightly offset by the weak Δ*E*_ads_ value of H_2_ (∼5 kJ mol^–1^). At the S_1/4 ML_-Fe­(110) surface, the energy barrier for H_2_ recombination was primarily controlled by the difference between the energy of reactants and products (Figure 5C). However, a small but broad peak was present along the reaction coordinate, corresponding to a local energy maximum where H_2_ desorption, accompanied by a displacement of S atoms, occurs. At the S_1/2 ML_-Fe­(110) surface, the recombination exhibited a significant reaction barrier of 70.8 kJ mol^–1^, despite a negligible difference in energy between the reactant and product geometries (Figure 5D). This barrier resulted from the unfavorable positioning of H* atoms near surface S atoms during the course of the reaction.

**Figure 5:**
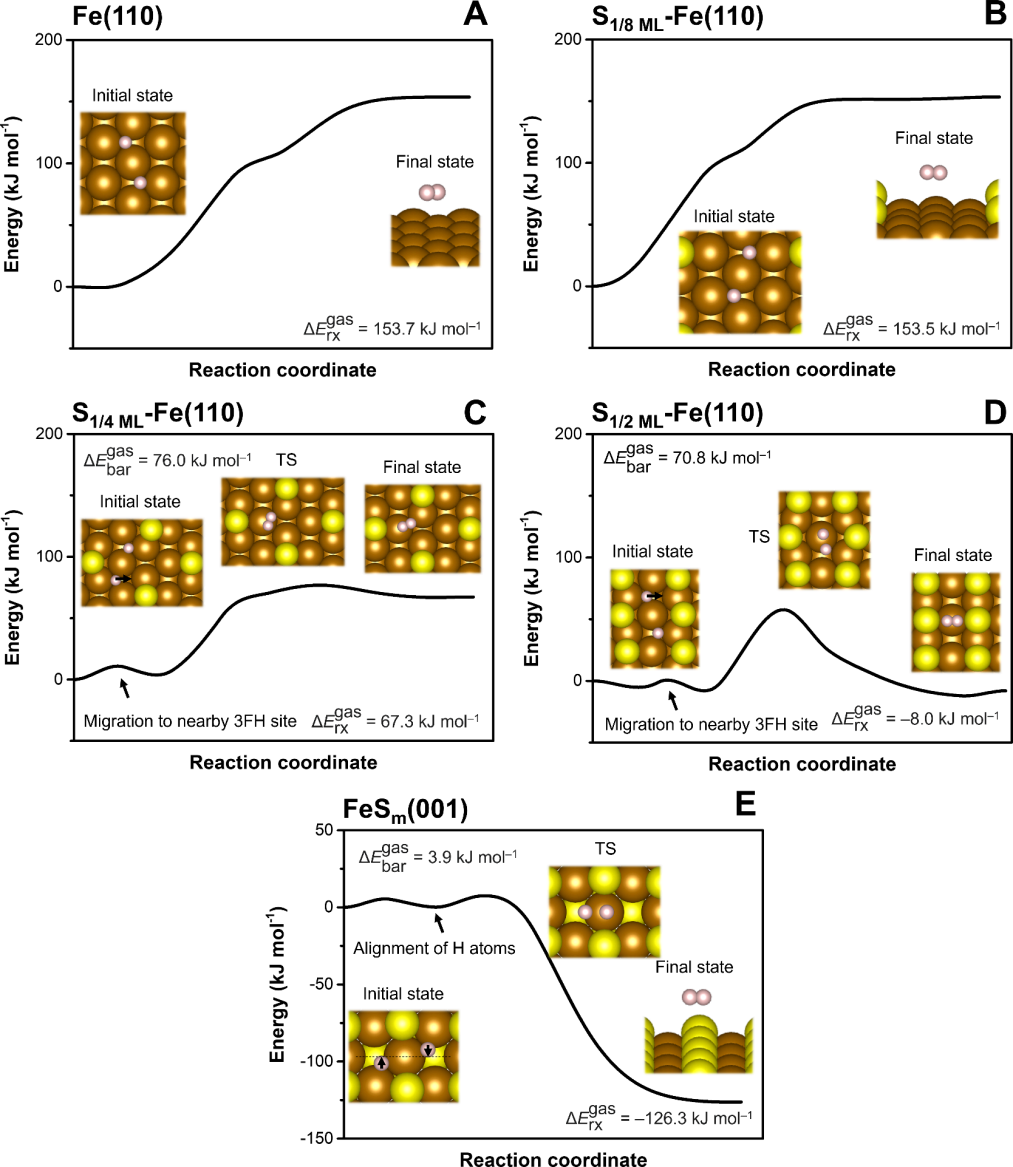
Reaction profiles of H*_ads_ recombination at pristine and sulfidated ZVI surfaces: (A) pristine Fe(110), (B) S_1/8 ML_-Fe­(110), (C) S_1/4 ML_-Fe­(110), (D) S_1/2 ML_-Fe­(110), and (E) FeS_m_(001). CI-NEB calculations were performed in the gas phase. The results of the transition state (TS) vibrational frequency analysis are shown in Table S8. The Δ*E*_bar_ values for (C–E) were calculated with respect to the optimized intermediate structure preceding the TS in the reaction coordinates (the H* migration before recombination is depicted by black arrows).

The observed increase in H*_ads_ recombination barriers at Fe(110) surfaces with moderately to high S coverage is consistent with the hypothesis proposed by Han and Yan that sulfidation hinders H*_ads_ recombination.^26^ Interestingly, at the FeS_m_(001) surface, H*_ads_ recombination exhibited only a minimal Δ*E*_bar_ (∼4 kJ mol^–1^) as a result of the highly positive Δ*E*_ads_ of H*_ads_ at this surface (Figure 5E). This reaction was strongly thermodynamically favorable with a reaction energy of −126.3 kJ mol^–1^.

Overall, these results indicate that H*_ads_ recombination at S-ZVI surfaces is mainly controlled by the favorability of H* adsorption, which decreases with increasing S coverage. At higher S coverage, recombination may be further hindered by unfavorable interactions between adsorbed S and H atoms along the reaction coordinate. Moreover, since the higher H*_ads_ migration barriers near S atoms (Figure 3) decrease the mobility of H*_ads_ at the S-ZVI surface, sulfidation also decreases the encounter frequency of H*_ads_, which effectively contributes to lowering its recombination rate.

### 3.6 Oxidation of ZVI Surface Promotes H* Recombination, Thus Limiting H* Availability

To assess the effects of surface corrosion on the feasibility of H*_ads_ recombination, we also calculated the profiles of Tafel reactions at ZVI surfaces with moderate O and OH coverages (Figure 6). The energy profiles of these reactions were comparable to the H*_ads_ recombination at the moderately sulfidated S_1/4 ML_-Fe­(110) surface (Figure 5C), albeit with higher Δ*E*_bar_ and Δ*E*_rx_ values attributable to the more favorable adsorption of H* at these surfaces, as discussed in previous sections. The flat central parts of the reaction coordinates suggest that H*_ads_ recombination is mainly controlled by the stability of H*_ads_ at the corroded ZVI surface. This indicates that during an ongoing surface corrosion, the formed H*_ads_ will gradually migrate to areas with a more favorable Δ*E*_ads_ or recombine to form H_2_.

**Figure 6:**
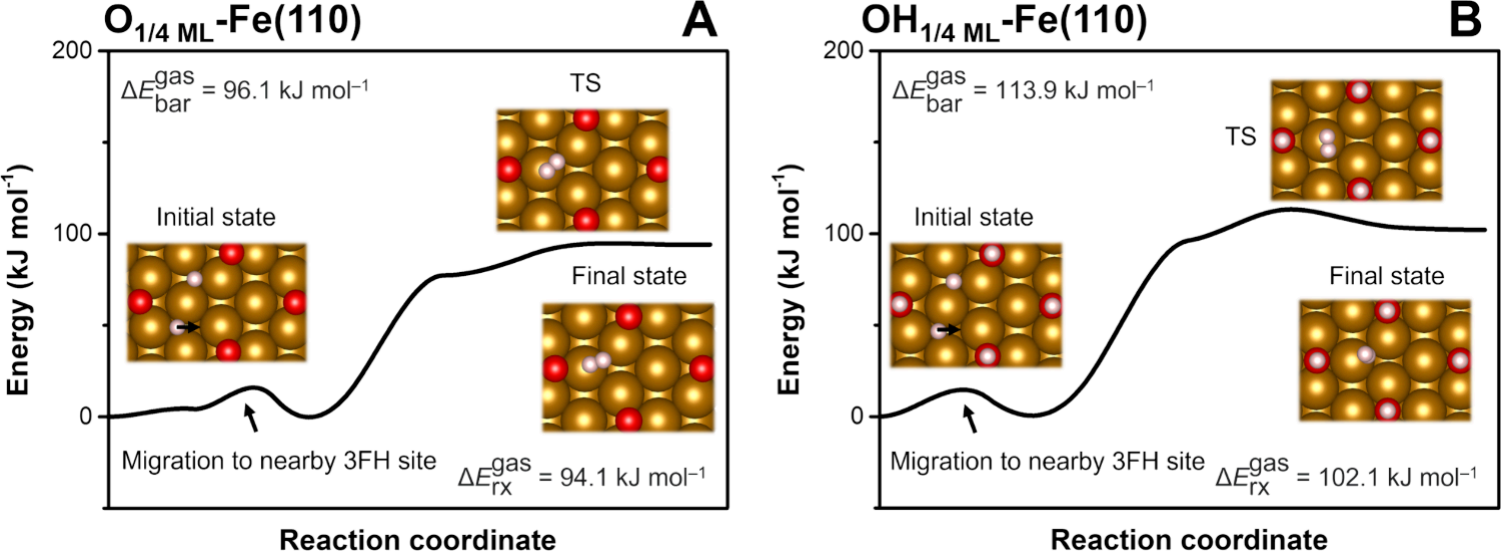
Reaction profiles of H*_ads_ recombination at moderately corroded ZVI surfaces: (A) O_1/4 ML_-Fe­(110) and (B) OH_1/4 ML_-Fe­(110). CI-NEB calculations were performed in the gas phase. The results of transition state (TS) vibrational frequency analysis are shown in Table S8. The Δ*E*_bar_ values were calculated with respect to the optimized intermediate structure preceding the TS in the reaction coordinates (the H* migration before recombination is depicted by black arrows).

### 3.7 Combined Effects of Sulfidation and Corrosion Control H*_ads_ Retention at the S-ZVI Surface

Our findings, complemented by the cited theoretical and experimental literature, offer a plausible mechanistic explanation for the increased retention of H*_ads_ at S-ZVI surfaces with moderate S coverage (Figure 7) observed experimentally.^32^

**Figure 7:**
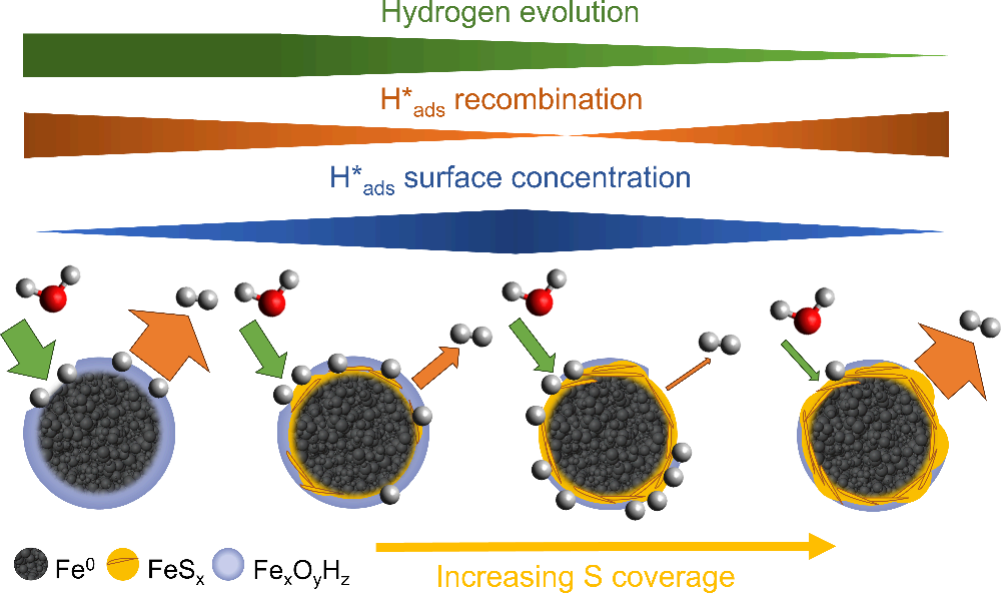
Proposed effects of sulfidation on H*_ads_ formation and fate at the S-ZVI surface. The widths of bands (top) and arrows (bottom) illustrate the rates of hydrogen evolution (green), H*_ads_ recombination (orange), and the extent of H*_ads_ retention at the particle surface (blue).

The pristine zero-valent Fe surface possesses a high abundance of T sites favorable for water adsorption and dissociation and 3FH sites favorable for H* adsorption. Yet, as its corrosion in water environments rapidly proceeds, the adsorbed O/OH species decrease the availability and accessibility of 3FH sites, and the H* adsorption on the corroded surface becomes less favorable. This ultimately promotes H* recombination, resulting in low H*_ads_ abundance at the ZVI surface in real scenarios.

The presence of S atoms at the ZVI surface induces two distinct effects: (i) it decreases the surface corrosion rate due to steric hindrance and the hydrophobic effect of S atoms, which also slows down the rate of H*_ads_ formation; and (ii) up to an S coverage of roughly 14 monolayer, it protects the available 3FH sites favorable for H* adsorption from corrosion, thus enhancing H*_ads_ retention. These binding sites can, in principle, also accommodate H*_ads_ migrating from areas of more intensive surface corrosion owing to their relatively low H*_ads_ migration barriers. This explains why S-ZVI particles with moderate S loading were experimentally observed to retain H*_ads_.^32^ Such accumulation of H*_ads_ is also favored by the increased barrier for H*_ads_ recombination at these surfaces. At higher S coverages, the corrosion is even more inhibited, and H* adsorption becomes thermodynamically less favorable, but S atoms may still hinder H*_ads_ recombination. After reaching critical S coverage, H*_ads_ recombination becomes both thermodynamically and kinetically favorable. These findings indicate that there is an optimal S coverage that maximizes the surface retention of H*_ads_. Resulting from our theoretical investigations, this optimal coverage can be close to 14 monolayer. At lower S coverages, the excessive corrosion will decrease the availability of 3FH sites, while at higher S coverages, the H*_ads_ recombination will proceed faster than H*_ads_ formation.

These findings have important implications for the reactivity of S-ZVI systems with contaminants. Particles with a higher H*_ads_ retention are especially convenient for the selective degradation of contaminants that undergo H*-mediated reductions, such as CEs with lower degrees of chlorination. Our results agree with the study by Mo and co-workers,^31^ who documented more pronounced S-induced reactivity improvement in vinyl chloride and *cis*-1,2-dichloroethene degradation using S-ZVI nanoparticles with a relatively low S/Fe molar ratio of 0.007 compared to more highly chlorinated CEs. This difference was attributed to the greater role of H*-mediated hydrogenolysis in the degradation of less chlorinated CEs. With recent advances in tailoring sulfur loading and speciation in S-ZVI synthesis,^10,70^ preparing materials with optimal H*_ads_ retention has become technically simple. This approach has great potential to further enhance the performance of S-ZVI in H*_ads_-mediated reductive destruction of contaminants, without the need to functionalize ZVI particles with expensive and environmentally problematic catalytic metals.^71^

It should be noted that the S-ZVI surface, including low S coverage, contains highly reactive sites for direct electron transfer as examined in detail in our previous study.^36^ Hence, the relative importance of direct electron transfer vs H*_ads_-mediated reactions and the absolute enhancement ratio (sulfidated vs nonsulfidated ZVI) at these surfaces will depend not only on S coverage but also other factors, including (i) the rate of H*_ads_ formation, which is controlled by pH,^62^ the presence of solutes,^4,63^ and phase composition of the outermost ZVI surface;^72^ (ii) the rate of H*_ads_ migration across varying surface morphologies; (iii) structural and steric parameters of the active site; and (iv) the nature of contaminants undergoing reduction.

### 3.8 Assumptions and Limitations of This Study

Several assumptions and limitations of this computational study should be acknowledged, most of which have already been discussed in detail in our prior works.^23,36^ First, the use of the straight Fe(110) surface as a model does not encompass all possible structural features present on the surface of S-ZVI, such as vacancies, steps, or kinks. These sites, with lower Fe atom coordination numbers, could exhibit higher reactivity in reactions with water and contaminants.^34^ However, it has been recently shown that sulfur has a high affinity for these highly reactive sites,^35^ which is in agreement with earlier evidence showing the deactivation of step edges by S atoms on Ni surfaces.^73^

Second, the S-doped surface models employed in this study were chosen as representatives of S-ZVI materials prepared by the postsulfidation method. Accurate atomic representation of the structure of S-ZVI prepared by the one-pot method would be much more challenging to construct due to the presence of various Fe^0^, S^0^, and FeS_*x*_ phases in the entire particle volume and nonuniform particle morphology.^9,22^ Nevertheless, it is reasonable to assume that the effect of S abundance on the behavior of H*_ads_ described here will also be relevant for S-ZVI prepared by the one-pot method.

Third, the S_1/8 ML_-Fe­(110), S_1/4 ML_-Fe­(110), and S_1/2 ML_-Fe­(110) models provide only simplified representations of the S-ZVI surface, as they neglect the presence of iron corrosion products on the particle surface, the role of which is discussed in separate sections using analogical models with S atoms replaced by O and OH. Furthermore, the postsulfidation method does not produce a perfectly uniform monolayer of S atoms, but the S atoms are distributed within a depth of several nm from the particle surface in poorly ordered phases,^21^ resulting in smaller effective S coverage on the particle topmost surface.

Lastly, this study does not include explicit water molecules as a solvent due to the complexity and computational cost of such calculations. Instead, we employed an implicit solvent model in the calculations of water adsorption and dissociation, representing the solvent as a polarizable continuum with the dielectric properties of water. This approximation does not account for specific and directional solvent-adsorbate or solvent-surface interactions such as hydrogen bonding. While this may reduce the absolute accuracy of the calculated energies, the relative trends reported here should remain reliable across the compared systems.

## 4 Conclusions

This DFT study investigates the effects of sulfidation and corrosion on the formation, stability, mobility, and recombination of H* at the ZVI surface. To this end, a series of Fe(110) slab model surfaces with increasing S coverage was employed, as well as their O-doped and OH-doped analogs. Moreover, pristine mackinawite (001) and amakinite (001) slab models were constructed to examine the stability of H* in typical primary products of ZVI sulfidation and corrosion. Our calculations reveal that sulfidation suppresses water adsorption and dissociation, while ZVI oxidation promotes further corrosion. Interestingly, both sulfidation and corrosion weaken H* adsorption affinity on the ZVI surface, promoting the migration of H* to more favorable adsorption sites or its recombination to form H_2_. As S-ZVI with moderate S coverage retains more reduced Fe sites favorable for H* adsorption compared to the corroded ZVI surface due to its higher corrosion resistance, this surface is prone to the accumulation of H*. Moreover, adsorbed H* exhibits restricted mobility near sulfur atoms, limiting their recombination and increasing their availability for contaminant degradation.

Our findings provide a plausible mechanistic explanation for the experimentally observed increase in H*_ads_ retention at the S-ZVI surfaces with moderate S coverage. Given the technical simplicity of tailoring sulfur loading in S-ZVI, this study establishes a basis for designing materials with optimized H*_ads_ retention. Such rational design can enhance the performance of S-ZVI in H*_ads_-mediated reductive degradation of contaminants, offering a cost-effective and environmentally friendly alternative to expensive and potentially harmful catalytic metals, while expanding the application potential of S-ZVI for groundwater remediation.

## Supplementary Material



## Data Availability

The data from the DFT calculations underlying this study are openly available in Zenodo at https://zenodo.org/records/17293091.

## Notes

Overview of surface slab models used in this study; DFT+D3-calculated adsorption energies of water molecule at the modeled surfaces; configurations of adsorbed water molecule at modeled surfaces with geometric parameters; imaginary vibrational modes and their frequencies for transition states of water dissociation; DFT+D3-calculated adsorption energies of H* at various sites of the modeled surfaces and H* migration energy barriers between these sites; horizontal displacement of S atoms in the S_1/8 ML_-Fe­(110) surface model upon H* adsorption to varying sites; charge density redistribution on the Fe(110) surface induced by S atoms in the S_1/8 ML_-Fe­(110) surface model; projected density of electronic states of the topmost Fe 3*d* electrons on the Fe surfaces doped with an increasing number of S atoms; nearest topmost S–S/O–O distances, surface density of H* adsorption sites, their connectivity, and H*_ads_ migration feasibility at pristine and S/O/OH-containing surfaces; effect of hydration on the stabilization and charge of H* adsorbed at varying sites at the S_1/8 ML_-Fe­(110) surface; and energy profile of H*_ads_ migration at the Fe­(OH)_2_(001) surface (PDF)

## References

[1] Bardos P., Merly C., Kvapil P., Koschitzky H.-P. (2018). Status
of Nanoremediation and Its Potential for Future Deployment: Risk-Benefit
and Benchmarking Appraisals. Remediat. J..

[2] Stefaniuk M., Oleszczuk P., Ok Y. S. (2016). Review on Nano Zerovalent Iron (NZVI):
From Synthesis to Environmental Applications. Chem. Eng. J..

[3] O’Carroll D., Sleep B., Krol M., Boparai H., Kocur C. (2013). Nanoscale
Zero Valent Iron and Bimetallic Particles for Contaminated Site Remediation. Adv. Water Resour..

[4] Liu Y., Phenrat T., Lowry G. V. (2007). Effect
of TCE Concentration and Dissolved
Groundwater Solutes on NZVI-Promoted TCE Dechlorination and H 2 Evolution. Environ. Sci. Technol..

[5] Schöftner P., Waldner G., Lottermoser W., Stöger-Pollach M., Freitag P., Reichenauer T. G. (2015). Electron
Efficiency of NZVI Does
Not Change with Variation of Environmental Parameters. Sci. Total Environ..

[6] Fan D., Lan Y., Tratnyek P. G., Johnson R. L., Filip J., O’Carroll D. M., Nunez Garcia A., Agrawal A. (2017). Sulfidation of Iron-Based Materials:
A Review of Processes and Implications for Water Treatment and Remediation. Environ. Sci. Technol..

[7] Li J., Zhang X., Sun Y., Liang L., Pan B., Zhang W., Guan X. (2017). Advances in
Sulfidation of Zerovalent
Iron for Water Decontamination. Environ. Sci.
Technol..

[8] Garcia A. N., Zhang Y., Ghoshal S., He F., O’Carroll D. M. (2021). Recent
Advances in Sulfidated Zerovalent Iron for Contaminant Transformation. Environ. Sci. Technol..

[9] Xu J., Wang Y., Weng C., Bai W., Jiao Y., Kaegi R., Lowry G. V. (2019). Reactivity, Selectivity,
and Long-Term
Performance of Sulfidized Nanoscale Zerovalent Iron with Different
Properties. Environ. Sci. Technol..

[10] Xu J., Avellan A., Li H., Liu X., Noël V., Lou Z., Wang Y., Kaegi R., Henkelman G., Lowry G. V. (2020). Sulfur Loading and
Speciation Control the Hydrophobicity,
Electron Transfer, Reactivity, and Selectivity of Sulfidized Nanoscale
Zerovalent Iron. Adv. Mater..

[11] Li H., Yang W., Wu C., Xu J. (2021). Origin of the Hydrophobicity
of Sulfur-Containing Iron Surfaces. Phys. Chem.
Chem. Phys..

[12] Gu Y., Gong L., Qi J., Cai S., Tu W., He F. (2019). Sulfidation Mitigates the Passivation of Zero Valent Iron at Alkaline
PHs: Experimental Evidences and Mechanism. Water
Res..

[13] Fan D., O’Brien
Johnson G., Tratnyek P. G., Johnson R. L. (2016). Sulfidation
of Nano Zerovalent Iron (NZVI) for Improved Selectivity During In-Situ
Chemical Reduction (ISCR). Environ. Sci. Technol..

[14] Gan S., Wang Z., Zheng C., Lin Z., Zhu A., Lai B. (2024). Enhanced Treatment
of Antimony Mine Wastewater by Sulfidated Micro
Zerovalent Iron (S-MZVI). Langmuir.

[15] Arnold W. A., Roberts A. L. (2000). Pathways and Kinetics of Chlorinated Ethylene and Chlorinated
Acetylene Reaction with Fe(0) Particles. Environ.
Sci. Technol..

[16] Elsner M., Chartrand M., VanStone N., Lacrampe Couloume G., Sherwood Lollar B. (2008). Identifying Abiotic Chlorinated Ethene Degradation:
Characteristic Isotope Patterns in Reaction Products with Nanoscale
Zero-Valent Iron. Environ. Sci. Technol..

[17] Wang J., Farrell J. (2003). Investigating the Role
of Atomic Hydrogen on Chloroethene
Reactions with Iron Using Tafel Analysis and Electrochemical Impedance
Spectroscopy. Environ. Sci. Technol..

[18] Li T., Farrell J. (2000). Reductive Dechlorination
of Trichloroethene and Carbon
Tetrachloride Using Iron and Palladized-Iron Cathodes. Environ. Sci. Technol..

[19] Xu J., Avellan A., Li H., Clark E. A., Henkelman G., Kaegi R., Lowry G. V. (2020). Iron and
Sulfur Precursors Affect
Crystalline Structure, Speciation, and Reactivity of Sulfidized Nanoscale
Zerovalent Iron. Environ. Sci. Technol..

[20] Xu J., Cao Z., Zhou H., Lou Z., Wang Y., Xu X., Lowry G. V. (2019). Sulfur Dose and Sulfidation Time Affect Reactivity
and Selectivity of Post-Sulfidized Nanoscale Zerovalent Iron. Environ. Sci. Technol..

[21] Brumovský M., Filip J., Malina O., Oborná J., Sracek O., Reichenauer T. G., Andrýsková P., Zbořil R. (2020). Core-Shell
Fe/FeS Nanoparticles with Controlled Shell
Thickness for Enhanced Trichloroethylene Removal. ACS Appl. Mater. Interfaces.

[22] Bhattacharjee S., Ghoshal S. (2018). Optimal Design of Sulfidated
Nanoscale Zerovalent Iron
for Enhanced Trichloroethene Degradation. Environ.
Sci. Technol..

[23] Brumovský M., Tunega D. (2023). Intrinsic Effects of Sulfidation on the Reactivity
of Zero-Valent Iron With Trichloroethene: A DFT Study. J. Phys. Chem. C.

[24] Cao Z., Xu J., Li H., Ma T., Lou L., Henkelman G., Xu X. (2020). Dechlorination and
Defluorination Capability of Sulfidized Nanoscale
Zerovalent Iron with Suppressed Water Reactivity. Chem. Eng. J..

[25] Cao Z., Li H., Xu X., Xu J. (2020). Correlating Surface
Chemistry and
Hydrophobicity of Sulfidized Nanoscale Zerovalent Iron with Its Reactivity
and Selectivity for Denitration and Dechlorination. Chem. Eng. J..

[26] Han Y., Yan W. (2016). Reductive Dechlorination of Trichloroethene by Zero-Valent
Iron Nanoparticles:
Reactivity Enhancement through Sulfidation Treatment. Environ. Sci. Technol..

[27] Gu Y., Wang B., He F., Bradley M. J., Tratnyek P. G. (2017). Mechanochemically
Sulfidated Microscale Zero Valent Iron: Pathways, Kinetics, Mechanism,
and Efficiency of Trichloroethylene Dechlorination. Environ. Sci. Technol..

[28] Mangayayam M. C., Perez J. P. H., Alonso-de-Linaje V., Dideriksen K., Benning L. G., Tobler D. J. (2022). Sulfidation Extent
of Nanoscale Zerovalent
Iron Controls Selectivity and Reactivity with Mixed Chlorinated Hydrocarbons
in Natural Groundwater. J. Hazard. Mater..

[29] Zhang Y., Ozcer P., Ghoshal S. (2021). A Comprehensive
Assessment of the
Degradation of C1 and C2 Chlorinated Hydrocarbons by Sulfidated Nanoscale
Zerovalent Iron. Water Res..

[30] Brumovský M., Micić V., Oborná J., Filip J., Hofmann T., Tunega D. (2023). Iron Nitride
Nanoparticles for Rapid Dechlorination
of Mixed Chlorinated Ethene Contamination. J.
Hazard. Mater..

[31] Mo Y., Xu J., Zhu L. (2022). Molecular Structure and Sulfur Content
Affect Reductive
Dechlorination of Chlorinated Ethenes by Sulfidized Nanoscale Zerovalent
Iron. Environ. Sci. Technol..

[32] Zhou G.-N., Chen J.-Q., Li W.-Q., He C.-S., Gong L., Liu X.-C., Wang Y.-R., Huang D., Mu Y. (2023). Enhanced Retention
of Surface-Adsorbed Atomic Hydrogen through Sulfidation of Nano Zerovalent
Iron for Water Decontamination. Chem. Eng. J..

[33] Filip J., Karlický F., Marušák Z., Lazar P., Černík M., Otyepka M., Zbořil R. (2014). Anaerobic
Reaction of Nanoscale Zerovalent Iron with Water: Mechanism and Kinetics. J. Phys. Chem. C.

[34] White J. J., Hinsch J. J., Bennett W. W., Wang Y. (2022). Theoretical Understanding
of Water Adsorption on Stepped Iron Surfaces. Appl. Surf. Sci..

[35] White J. J., Hinsch J. J., Wu Z., Tian Y., Bennett W. W., Wang Y. (2023). Sulfidation Impacts on the Hydrophobicity
of Stepped Iron Surfaces. Adv. Energy Sustainability
Res..

[36] Brumovský M., Tunega D. (2024). Reductive Dechlorination of Chlorinated Ethenes at
the Sulfidated Zero-Valent Iron Surface: A Mechanistic DFT Study. J. Phys. Chem. C.

[37] White J. J., Zhou M., Hinsch J. J., Bennett W. W., Wang Y. (2024). A Theoretical
Investigation on Sulfidated Nanoscale Zero Valent Iron for Removal
of Cis-DCE and PCE. Microstructures.

[38] Kresse G., Hafner J. (1993). Ab Initio Molecular
Dynamics for Open-Shell Transition
Metals. Phys. Rev. B.

[39] Kresse G., Furthmüller J. (1996). Efficient
Iterative Schemes for Ab Initio Total-Energy
Calculations Using a Plane-Wave Basis Set. Phys.
Rev. B.

[40] Kresse G., Furthmüller J. (1996). Efficiency of Ab-Initio Total Energy
Calculations for
Metals and Semiconductors Using a Plane-Wave Basis Set. Comput. Mater. Sci..

[41] Blöchl P. E. (1994). Projector
Augmented-Wave Method. Phys. Rev. B.

[42] Kresse G., Joubert D. (1999). From Ultrasoft Pseudopotentials to the Projector Augmented-Wave
Method. Phys. Rev. B.

[43] Perdew J. P., Burke K., Ernzerhof M. (1996). Generalized
Gradient Approximation
Made Simple. Phys. Rev. Lett..

[44] Grimme S., Ehrlich S., Goerigk L. (2011). Effect of the Damping Function in
Dispersion Corrected Density Functional Theory. J. Comput. Chem..

[45] Grimme S., Antony J., Ehrlich S., Krieg H. (2010). A Consistent and Accurate
Ab Initio Parametrization of Density Functional Dispersion Correction
(DFT-D) for the 94 Elements H-Pu. J. Chem. Phys..

[46] Monkhorst H. J., Pack J. D. (1976). Special Points for
Brillouin-Zone Integrations. Phys. Rev. B.

[47] Tunega D. (2012). Theoretical
Study of Properties of Goethite (α-FeOOH) at Ambient and High-Pressure
Conditions. J. Phys. Chem. C.

[48] Rollmann G., Rohrbach A., Entel P., Hafner J. (2004). First-Principles Calculation
of the Structure and Magnetic Phases of Hematite. Phys. Rev. B.

[49] Henkelman G., Uberuaga B. P., Jónsson H. (2000). A Climbing
Image Nudged Elastic Band
Method for Finding Saddle Points and Minimum Energy Paths. J. Chem. Phys..

[50] Henkelman G., Jónsson H. (1999). A Dimer Method
for Finding Saddle Points on High Dimensional
Potential Surfaces Using Only First Derivatives. J. Chem. Phys..

[51] Mathew K., Sundararaman R., Letchworth-Weaver K., Arias T. A., Hennig R. G. (2014). Implicit
Solvation Model for Density-Functional Study of Nanocrystal Surfaces
and Reaction Pathways. J. Chem. Phys..

[52] Mathew K., Kolluru V. S. C., Mula S., Steinmann S. N., Hennig R. G. (2019). Implicit Self-Consistent Electrolyte
Model in Plane-Wave
Density-Functional Theory. J. Chem. Phys..

[53] Błoński P., Kiejna A. (2007). Structural,
Electronic, and Magnetic Properties of
Bcc Iron Surfaces. Surf. Sci..

[54] Brumovský M., Oborná J., Micić V., Malina O., Kašlík J., Tunega D., Kolos M., Hofmann T., Karlický F., Filip J. (2022). Iron Nitride Nanoparticles for Enhanced Reductive Dechlorination
of Trichloroethylene. Environ. Sci. Technol..

[55] Kolos M., Tunega D., Karlický F. (2020). A Theoretical Study of Adsorption
on Iron Sulfides towards Nanoparticle Modeling. Phys. Chem. Chem. Phys..

[56] Liu S., Tian X., Wang T., Wen X., Li Y.-W., Wang J., Jiao H. (2015). Coverage Dependent
Water Dissociative
Adsorption on Fe(110) from DFT Computation. Phys. Chem. Chem. Phys..

[57] Wyckoff, R. W. G. Crystal Structures - Vol. 1; Interscience Publishers: New York, USA, 1963.

[58] Lin Z., Xu J., Zhu A., He C., Wang C., Zheng C. (2023). Physicochemical
Effects of Sulfur Precursors on Sulfidated Amorphous Zero-Valent Iron
and Its Enhanced Mechanisms for Cr­(VI) Removal. Langmuir.

[59] Dzade N. Y., Roldan A., de Leeuw N. H. (2016). DFT-D2 Simulations
of Water Adsorption
and Dissociation on the Low-Index Surfaces of Mackinawite (FeS). J. Chem. Phys..

[60] Bae S., Collins R. N., Waite T. D., Hanna K. (2018). Advances in Surface
Passivation of Nanoscale Zerovalent Iron: A Critical Review. Environ. Sci. Technol..

[61] Liu S., Tian X., Wang T., Wen X., Li Y.-W., Wang J., Jiao H. (2015). Coverage Dependent
Water Dissociative
Adsorption on the Clean and O-Precovered Fe(111) Surfaces. J. Phys. Chem. C.

[62] Liu Y., Lowry G. V. (2006). Effect of Particle
Age (Fe 0 Content) and Solution
PH On NZVI Reactivity: H 2 Evolution and TCE Dechlorination. Environ. Sci. Technol..

[63] Turcio-Ortega D., Fan D., Tratnyek P. G., Kim E.-J., Chang Y.-S. (2012). Reactivity of Fe/FeS
Nanoparticles: Electrolyte Composition Effects on Corrosion Electrochemistry. Environ. Sci. Technol..

[64] Mangayayam M. C., Alonso-de-Linaje V., Dideriksen K., Tobler D. J. (2020). Effects of Common
Groundwater Ions on the Transformation and Reactivity of Sulfidized
Nanoscale Zerovalent Iron. Chemosphere.

[65] Eder M., Terakura K., Hafner J. (2001). Initial Stages
of Oxidation of (100)
and (110) Surfaces of Iron Caused by Water. Phys. Rev. B.

[66] Jiang D., Carter E. A. (2003). Adsorption and Diffusion Energetics
of Hydrogen Atoms
on Fe(110) from First Principles. Surf. Sci..

[67] Jeong H. Y., Hayes K. F. (2007). Reductive Dechlorination of Tetrachloroethylene
and
Trichloroethylene by Mackinawite (FeS) in the Presence of Metals:
Reaction Rates. Environ. Sci. Technol..

[68] He F., Li Z., Shi S., Xu W., Sheng H., Gu Y., Jiang Y., Xi B. (2018). Dechlorination
of Excess Trichloroethene
by Bimetallic and Sulfidated Nanoscale Zero-Valent Iron. Environ. Sci. Technol..

[69] Odziemkowski M. S., Simpraga R. P. (2004). Distribution of
Oxides on Iron Materials Used for Remediation
of Organic Groundwater Contaminants & #151; Implications for Hydrogen
Evolution Reactions. Can. J. Chem..

[70] Xu, W. ; Xia, C. ; He, F. ; Wang, Z. ; Liang, L. Sulfidation of Nanoscale Zero-Valent Iron by Sulfide: The Dynamic Process, Mechanism, and Role of Ferrous Iron. Environ. Sci. Technol. 2024. 10.1021/acs.est.4c04390. 39262330

[71] Yang L., Zhou W., Zhang M. (2025). Intentional
Corrosion-Induced Asymmetric
Charge on Ru/NZVI-Ni for Efficient Atomic Hydrogen-Mediated Dechlorination:
Microsolvation Effects. Appl. Catal. B Environ.
Energy.

[72] Qin H., Guan X., Bandstra J. Z., Johnson R. L., Tratnyek P. G. (2018). Modeling
the Kinetics of Hydrogen Formation by Zerovalent Iron: Effects of
Sulfidation on Micro- and Nano-Scale Particles. Environ. Sci. Technol..

[73] Abild-Pedersen F., Lytken O., Engbæk J., Nielsen G., Chorkendorff I., No̷rskov J. K. (2005). Methane Activation on Ni(111): Effects of Poisons and
Step Defects. Surf. Sci..

