# Supplementary material for: Mechanism of Increased Retention of Atomic Hydrogen on Moderately Sulfidated Zero-Valent Iron Surfaces
Source: Langmuir. 2025 Oct 9;41(41):28193–206. doi: 10.1021/acs.langmuir.5c04136 (PMC12548089; doi:10.1021/acs.langmuir.5c04136)
Supplement: Supplementary file 1 [file la5c04136_si_001.pdf]

Supporting Information

Mechanism of Increased Retention of Atomic Hydrogen on Moderately Sulfidated Zero-Valent Iron Surfaces

Miroslav Brumovský [a,b] * *and Daniel Tunega* [b]

[a] Regional Centre of Advanced Technologies and Materials, Czech Advanced Technology and Research Institute (CATRIN), Palacký University Olomouc, Šlechtitelů 27, 779 00 Olomouc, Czech Republic

[b] University of Natural Resources and Life Sciences, Vienna, Department of Ecosystem Management, Climate and Biodiversity, Institute of Soil Research, Peter-Jordan-Straße 82, 1190 Vienna, Austria

* Corresponding author

E-mail address: miroslav.brumovsky@upol.cz

Summary

Number of pages: 12

Number of tables: 8

Number of figures: 9

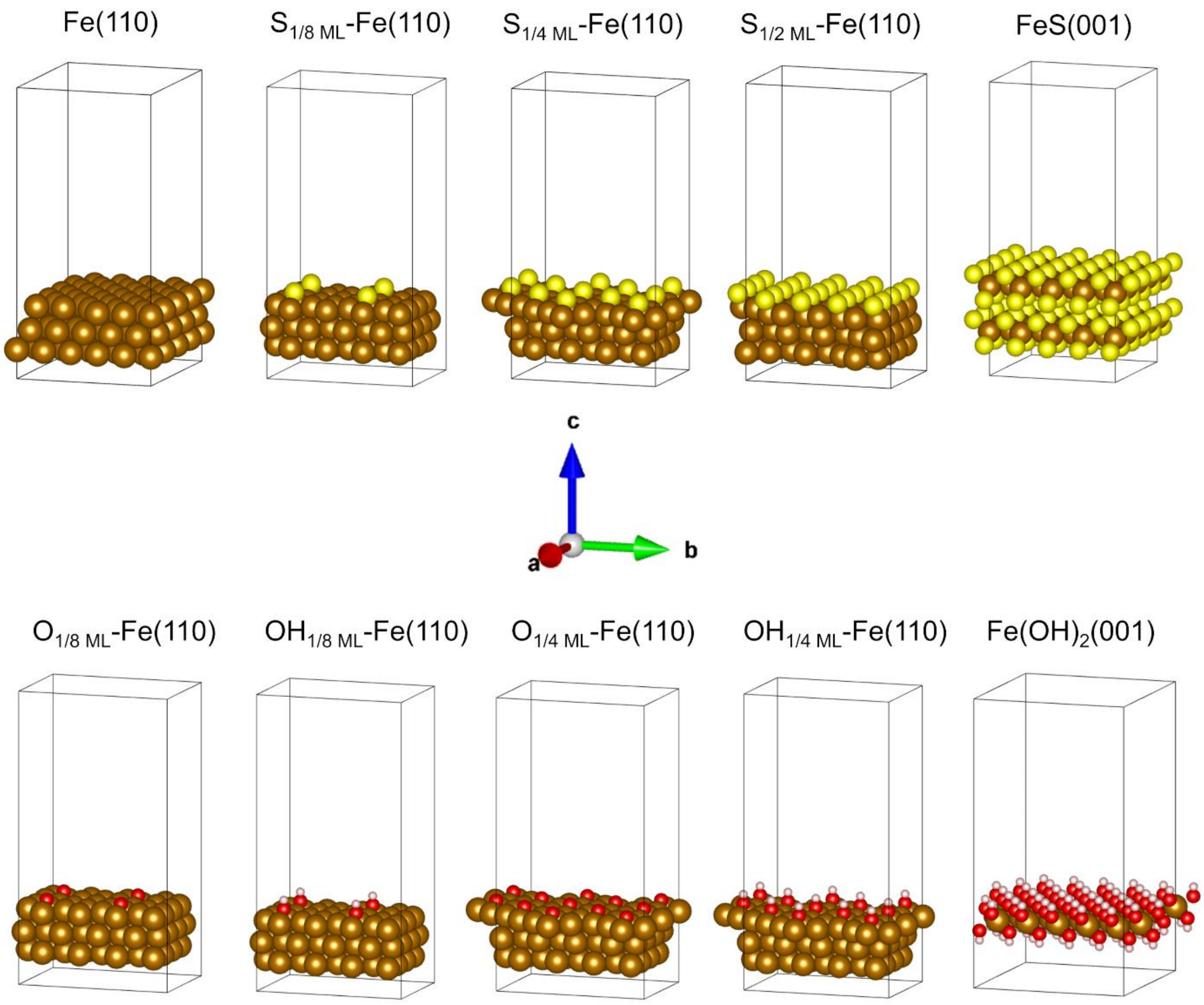

Figure S1. Overview of surface slab models used in this study (atom coloring: Fe (brown), S (yellow), O (red), and H (white)).

Table S1. DFT+D3 calculated adsorption energies of a water molecule at the modeled surfaces. Values were calculated in the gas phase and with an implicit solvent model (VASPsol).

**Adsorption complex**	**Energy (kJ mol^{-1})**	
	**Gas phase**	**Solvent**
Fe(110)···H_2O	–51.4	–59.0
$S_{1/8\ ML}$-Fe(110)···H_2O	–54.0	–56.3
$S_{1/4\ ML}$-Fe(110)···H_2O	–42.3	–23.5
$S_{1/2\ ML}$-Fe(110)···H_2O	–22.9	–13.7
FeS_m(001)···H_2O	–19.4	–8.5
$O_{1/8\ ML}$-Fe(110)···H_2O	–67.9	–60.2
$O_{1/4\ ML}$-Fe(110)···H_2O	–79.7	–62.0
$OH_{1/8\ ML}$-Fe(110)···H_2O (H-bond)	–62.8	–24.5
$OH_{1/8\ ML}$-Fe(110)···H_2O	–46.5	–31.8
$OH_{1/4\ ML}$-Fe(110)···H_2O (H-bond)	–55.2	–15.1
$Fe(OH)_2$(001)···H_2O (H-bond)	–42.8	–21.7

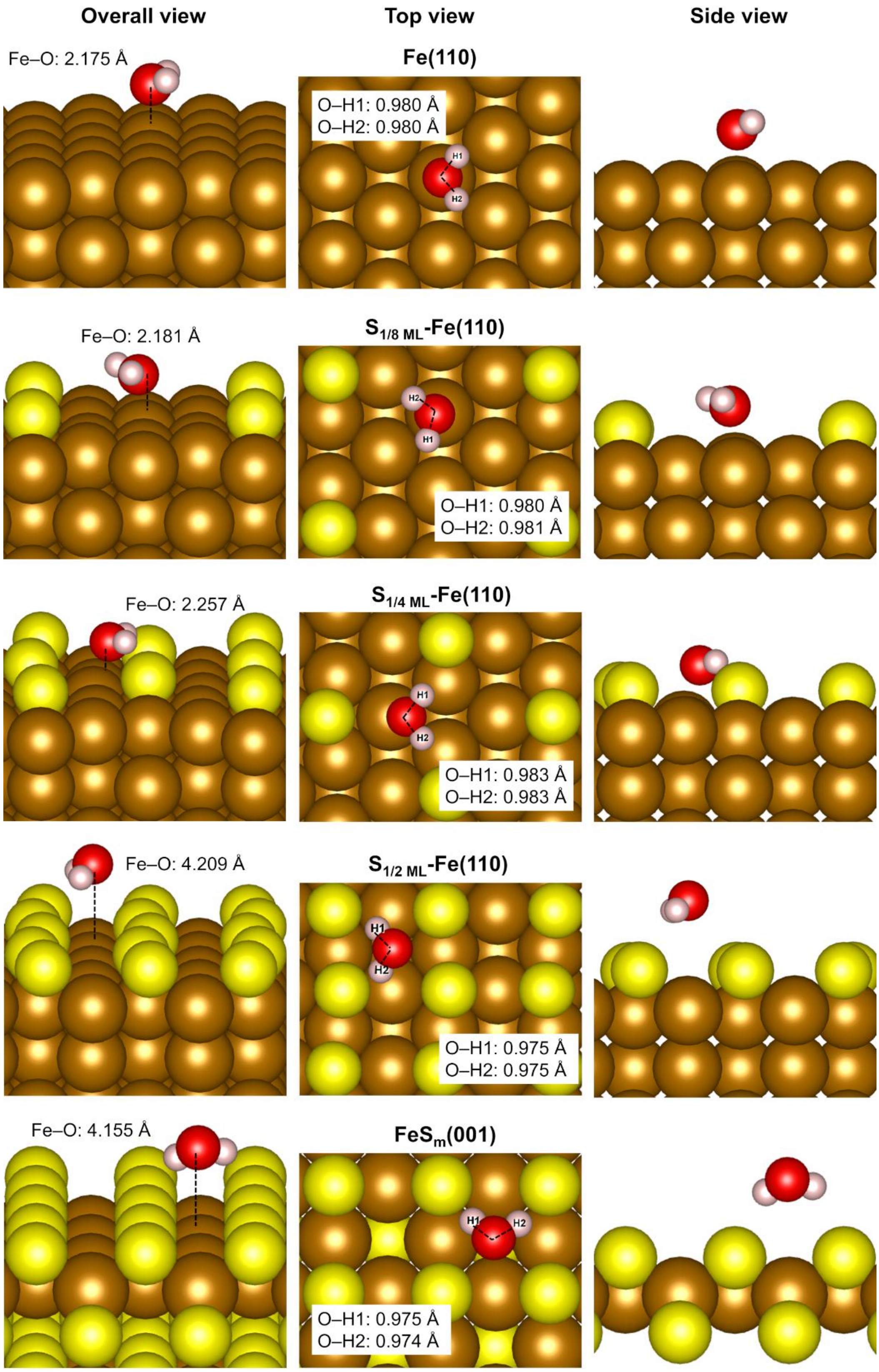

Figure S2. Overall, top, and side views of PBE+D3-optimized configurations of a water molecule adsorbed on Fe(110) surfaces with increasing S coverage and the $FeS_m(001)$ surface with shown Fe–O and O–H distances. Atom coloring: Fe (brown), S (yellow), O (red), and H (white).

Table S2. Imaginary vibrational modes and their frequencies for transition states of water dissociation.

Surface	Imaginary vibrational mode	Corresponding frequency (cm $^{-1}$)
Fe(110)	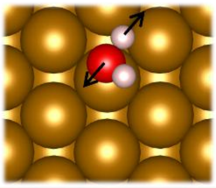	–1203
$S_{1/8\ ML}$-Fe(110)	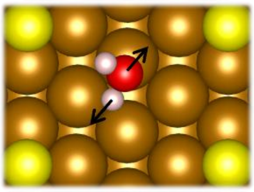	–1176
$S_{1/4\ ML}$-Fe(110)	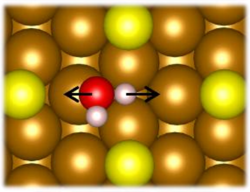	–1128
$S_{1/2\ ML}$-Fe(110)	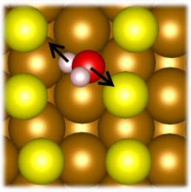	–167
FeS_m(001)	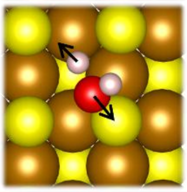	–184
$O_{1/4\ ML}$-Fe(110)	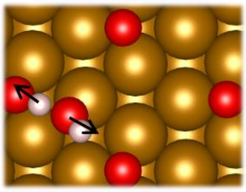	–597
$OH_{1/4\ ML}$-Fe(110)	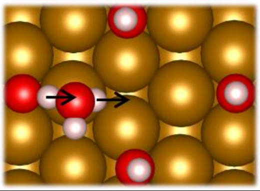	–1310

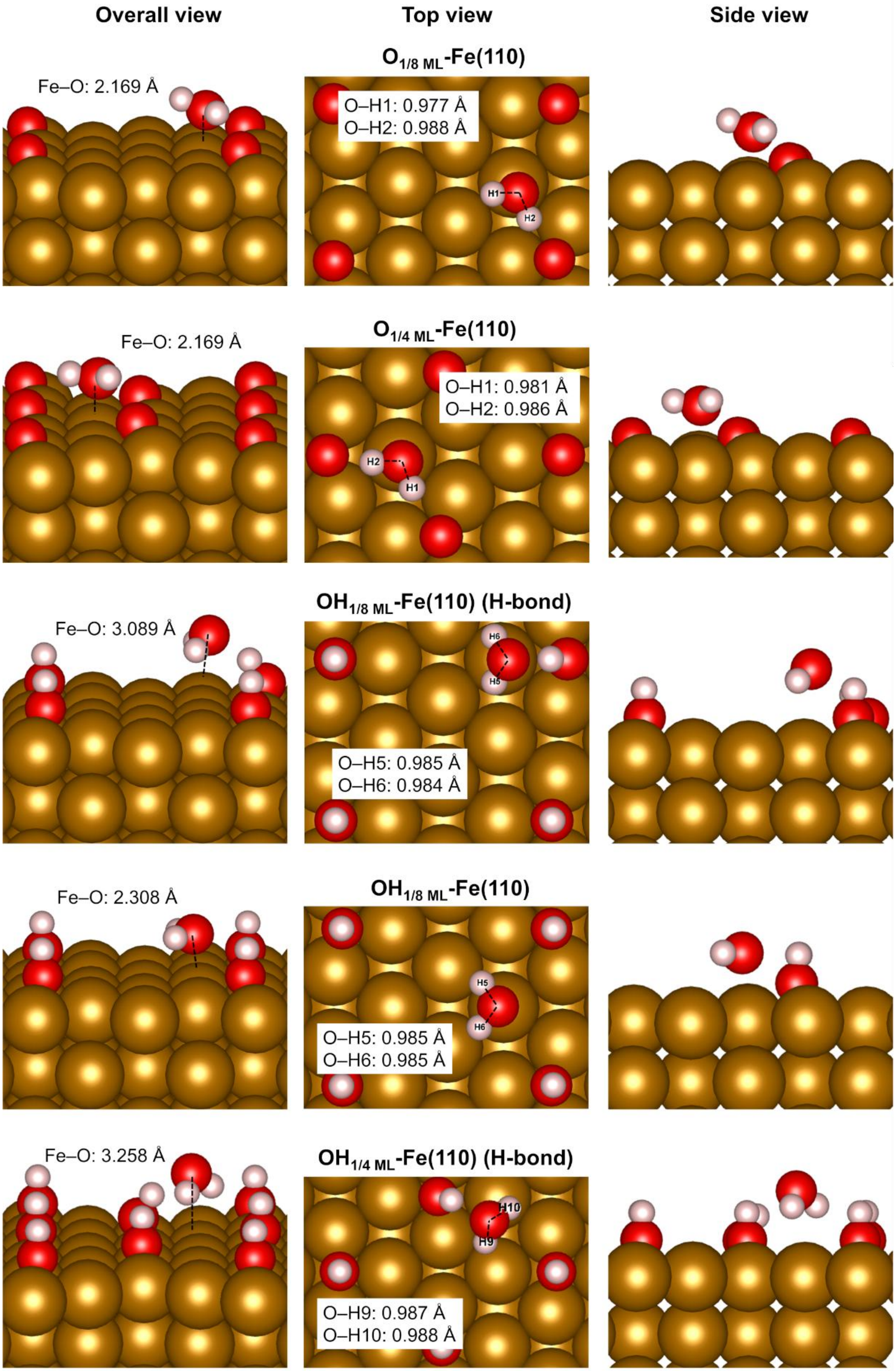

Figure S3. Overall, top, and side views of PBE+D3-optimized configurations of a water molecule adsorbed on Fe(110) surfaces with increasing O/OH coverage with shown Fe–O and O–H distances. Atom coloring: Fe (brown), O (red), and H (white).

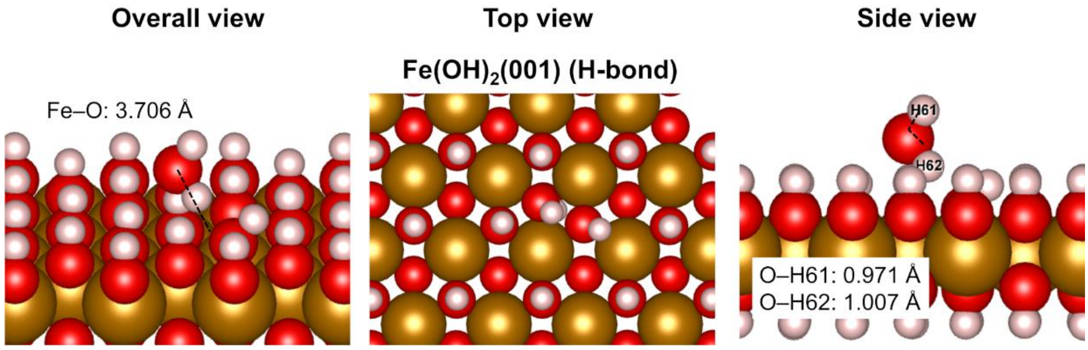

Figure S4. Overall, top, and side views of PBE+D3-optimized configurations of a water molecule adsorbed on the Fe(OH)$_2$(001) surface with shown Fe–O and O–H distances. Atom coloring: Fe (brown), O (red), and H (white).

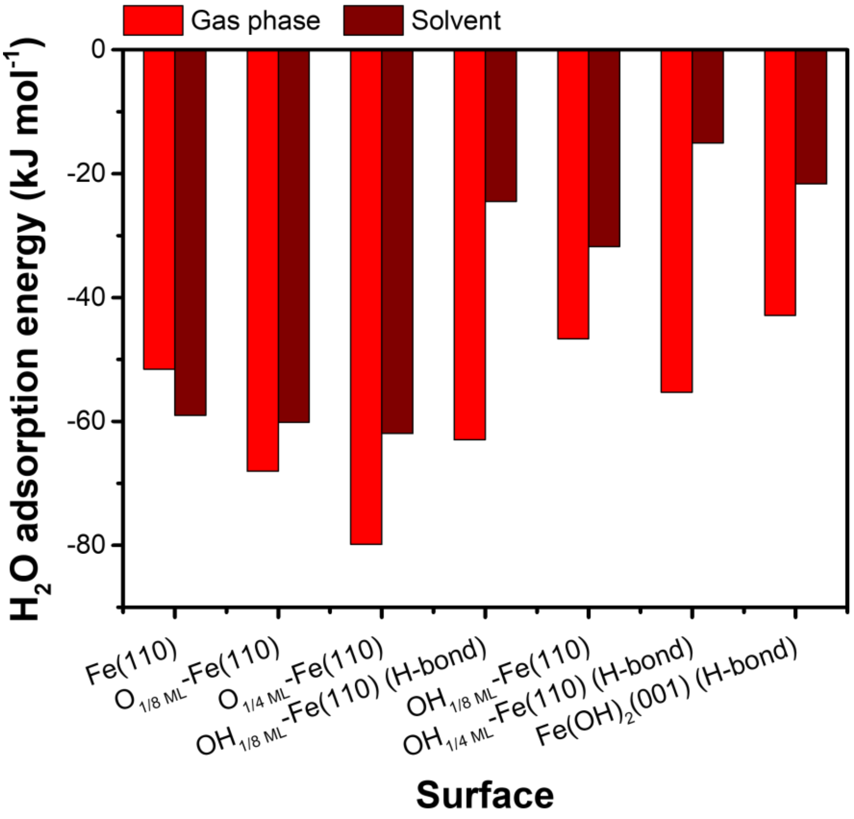

Figure S5. Water adsorption energies on the studied pristine and oxidized/hydroxylated Fe-bearing surfaces in the gas phase and in solvent (water).

Table S3. DFT+D3-calculated adsorption energies of H*$_{ads}$ at various sites of the pristine Fe(110), sulfidated Fe(110), and FeS$_m$(001) surfaces and energy barriers between them. Values were calculated in the gas phase. Further description of adsorption sites and the H*$_{ads}$ migration pathways is provided in Figure 3 in the manuscript.

Surface model	Site	H* adsorption energy (kJ mol^{-1})	H* migration direction	H* migration barrier (kJ mol^{-1})
Fe(110)	3FH	-78.8	3FH --> LB --> 3FH	5.4
	LB	-73.3	3FH --> SB --> 3FH	16.5
	SB	-62.2		
$S_{1/8\ ML}$-Fe(110)	3FH-A	-80.6	3FH-A --> 3FH-A	4.4
	3FH-B	-72.9	3FH-A --> 3FH-B	19.0
	3FH-C	-77.8	3FH-B --> 3FH-C	14.4
	3FH-D	-28.0	3FH-B --> 3FH-D	44.9
	3FH-E	-62.2	3FH-C --> 3FH-C	5.2
	T-S	78.5	3FH-D --> 3FH-E	7.0
			3FH-E --> 3FH-E	6.1
$S_{1/4\ ML}$-Fe(110)	3FH-A	-59.2	3FH-A --> 3FH-A	4.4
	3FH-B	-36.5	3FH-A --> 3FH-B	27.8
	T-S	79.7	3FH-B --> 3FH-B	10.8
$S_{1/2\ ML}$-Fe(110)	3FH-A	-1.1	3FH-A --> 3FH-A	11.0
	T-S	62.1	3FH-A --> 1 --> 3FH-A	98.7
			3FH-A --> 2 --> 3FH-A	59.3
FeS$_m$(001)	2FH-A	64.8	2FH-A --> 2FH-A	3.8
	T-S	77.2	2FH-A --> 1 --> 2FH-A	22.1

Table S4. Horizontal displacement of S atoms with respect to the underlying Fe atom in the $S_{1/8\ ML}$-Fe(110) surface model upon H* adsorption to varying sites. Note that the H* adsorption complex at the "D" site is not included as it was calculated with frozen geometry to avoid H* migration to the nearby "B" site.

	Displacement of S atoms in the adsorption complex (Å)				
**Site**	**S1**	**S2**	**S3**	**S4**	**Average**
3FH-A	0.009	0.009	0.001	0.001	0.005
3FH-B	0.004	0.103	0.003	0.004	0.028
3FH-C	0.002	0.037	0.029	0.007	0.019
3FH-E	0.089	0.089	0.002	0.002	0.045
T-S	0.005	0.005	0.001	0.001	0.003

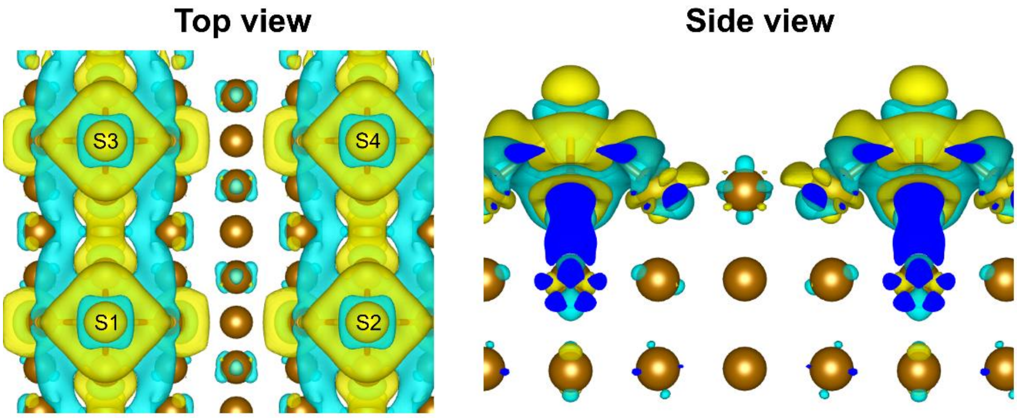

Figure S6. PBE-calculated charge density redistribution on the Fe(110) surface induced by four S atoms in the $S_{1/8\ ML}$-Fe(110) surface model. The yellow isosurface indicates an electron gain, while the blue one represents an electron loss. The isosurface level was set to 0.001 $Bohr^{-3}$.

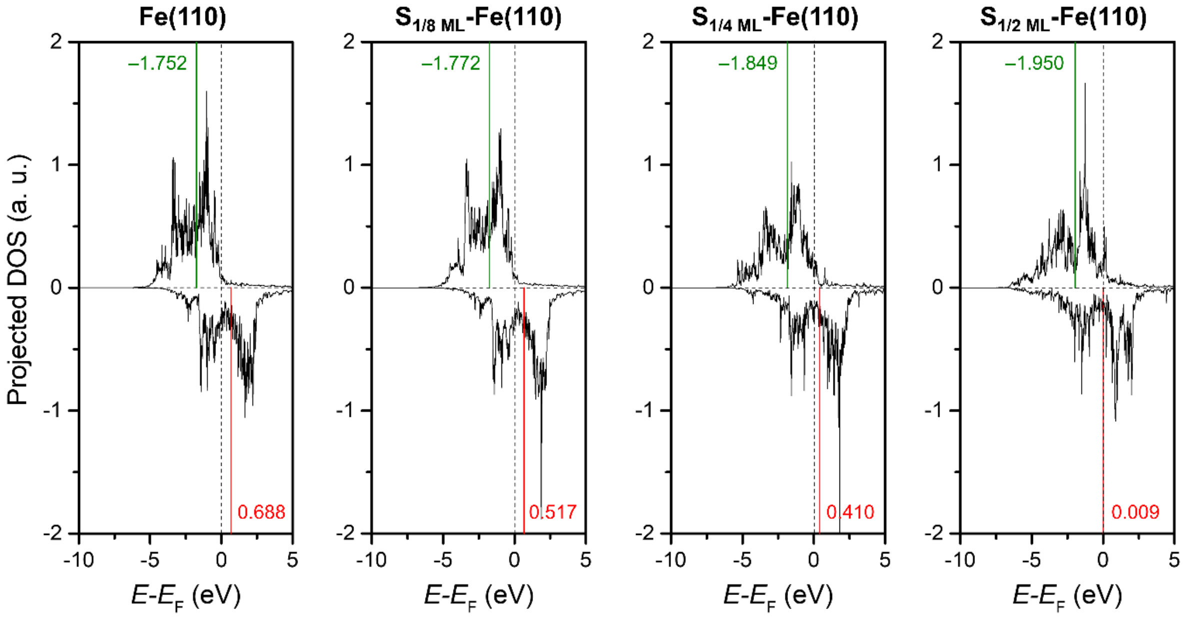

Figure S7. Projected density of electronic states of the topmost Fe 3*d* electrons on the Fe surfaces doped with an increasing number of S atoms (no adsorbate present). The positions of the *d*-band centers for spin up and down states are indicated in green and red, respectively. The *d*-band centers are referenced to the Fermi level (E_F = 0 eV). The DOS calculations were performed using the tetrahedron smearing method with Blöchl corrections.[1] The post-processing of DOS calculations was performed using the program VASPKIT.[2]

Table S5. Nearest topmost S-S distances, surface density of H* adsorption sites, their connectivity, and H^*_{ads} migration feasibility at pristine and sulfidated surfaces.

Surface model	Nearest S-S distance (Å)	H^*_{ads} sites per 100 Å^2*	Average site connectivity†	Free H^*_{ads} migration	
				Direction a	Direction b
Fe(110)	--	36.6 (36.6)	3	Yes	Yes
$S_{1/8\ ML}$-Fe(110)	5.50	22.9 (32.1)	2.2	No	Yes
$S_{1/4\ ML}$-Fe(110)	4.83	27.3 (27.3)	2.3	Yes (diagonal)	Yes (diagonal)
$S_{1/2\ ML}$-Fe(110)	2.89	0 (17.8)	1	No	No
FeS_m(001)	3.57	0 (31.4‡)	4	Yes§	Yes§

* Number of sites favorable for H* adsorption with ΔE_{ads} << 0 and stable H* adsorption configuration, in () number of total available adsorption sites.

† Weighted average of unrestricted H^*_{ads} migration pathways at room temperature (ΔE_{bar} < 30 kJ mol^{-1}) from all favorable adsorption sites.

‡ The greater total number of H* adsorption sites relative to the $S_{1/2\ ML}$–Fe(110) surface arises from the different surface symmetry, where each 3FH site effectively splits into two 2FH sites.

§ H^*_{ads} is not stable at this surface and will recombine to H_2.

Table S6. DFT+D3 calculated adsorption energies of H^*_{ads} at various sites of the O/OH-doped Fe(110) and $Fe(OH)_2$(001) surfaces and energy barriers between them. Values were calculated in the gas phase. Further description of adsorption sites and the H^*_{ads} migration pathways is provided in Figure 4 in the manuscript.

Surface model	Site	H* adsorption energy (kJ mol^{-1})	H* migration direction	H* migration barrier (kJ mol^{-1})
$O_{1/8\ ML}$-Fe(110)	3FH-A	-87.2	3FH-A --> 3FH-A	7.1
	3FH-B	-80.7	3FH-A --> 3FH-B	19.4
	3FH-C	-83.1	3FH-B --> 3FH-C	15.7
	3FH-D	-67.1	3FH-B --> 3FH-D	14.1
	3FH-E	-68.4	3FH-C --> 3FH-C	3.8
	T-O	-8.2	3FH-D --> 3FH-E	13.5
			3FH-E --> 3FH-E	16.0
$OH_{1/8\ ML}$-Fe(110)	3FH-A	-83.5	3FH-A --> 3FH-A	5.0
	3FH-B	-80.3	3FH-A --> 3FH-B	18.6
	3FH-C	-82.1	3FH-B --> 3FH-C	16.3
	3FH-D	-67.3	3FH-B --> 3FH-D	13.1
	3FH-E	-71.8	3FH-C --> 3FH-C	4.1
			3FH-D --> 3FH-E	13.2
			3FH-E --> 3FH-E	16.2
$O_{1/4\ ML}$-Fe(110)	3FH-A	-59.2	3FH-A --> 3FH-A	17.0
	3FH-B	-36.5	3FH-A --> 3FH-B	18.0
	T-O	79.7	3FH-B --> 3FH-B	11.6
$OH_{1/4\ ML}$-Fe(110)	3FH-A	-61.4	3FH-A --> 3FH-A	9.4
	3FH-B	-55.5	3FH-A --> 3FH-B	18.8
			3FH-B --> 3FH-B	3.8
$Fe(OH)_2$(001)	T-A	139.9	T-A --> T-A	n.d.*

* n.d. – not determined.

Table S7. Nearest topmost O-O distances, surface density of H* adsorption sites, their connectivity, and H^*_{ads} migration feasibility at oxidized and hydroxylated surfaces.

Surface model	Nearest O-O distance (Å)	H^*_{ads} sites per 100 $Å^2$*	Average site connectivity†	Free H^*_{ads} migration	
				a	b
$O_{1/8\,ML}$-Fe(110)	5.52	32.0 (32.0)	2.7	Yes	Yes
$OH_{1/8\,ML}$-Fe(110)	5.50	32.0 (32.0)	2.7	Yes	Yes
$O_{1/4\,ML}$-Fe(110)	4.83	27.4 (27.4)	2.3	Yes (diagonal)	Yes (diagonal)
$OH_{1/4\,ML}$-Fe(110)	4.86	27.1 (27.1)	2.3	Yes (diagonal)	Yes (diagonal)
$Fe(OH)_2$(001)	3.27	0 (10.8)	0‡	No	No

* Number of sites favorable for H* adsorption with ΔE_{ads} << 0 and stable H* adsorption configuration, in () number of total available adsorption sites.

† Weighted average of unrestricted H^*_{ads} migration pathways at room temperature (ΔE_{bar} < 30 kJ mol^{-1}) from all favorable adsorption sites.

‡ H^*_{ads} recombines to H_2 during migration (Figure S9).

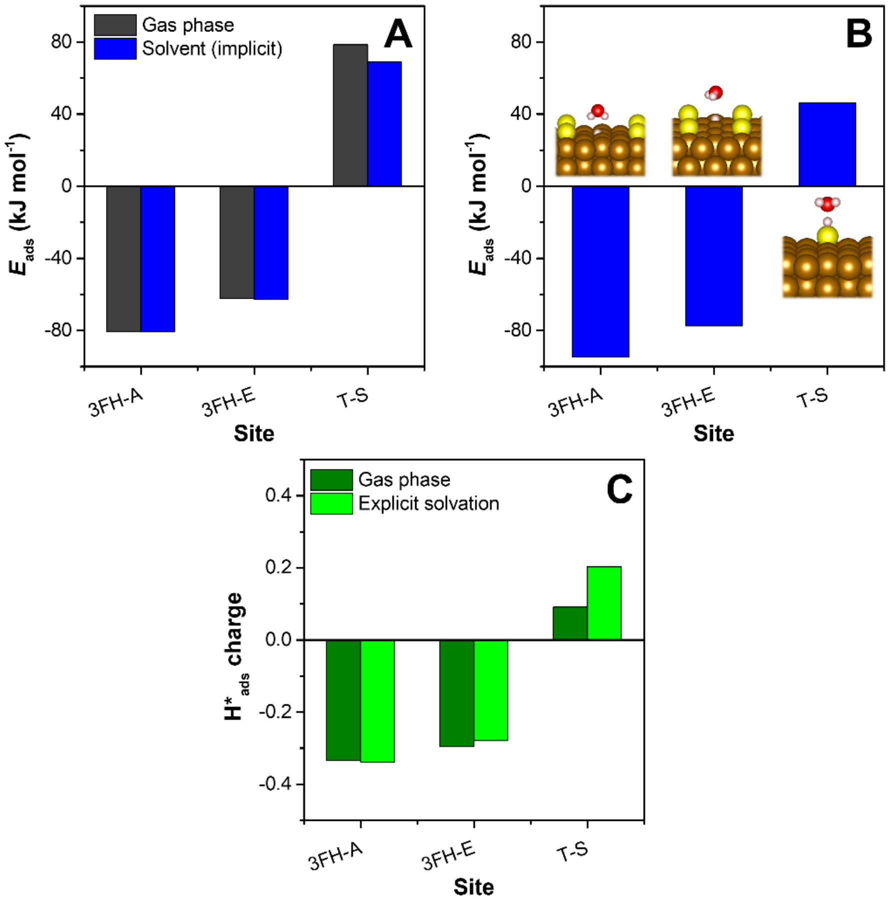

Figure S8. The effect of hydration on the stabilization and charge of H* adsorbed at varying sites at the $S_{1/8\,ML}$-Fe(110) surface: (A) implicit solvation using the continuum solvation model VASPsol; (B) explicit solvation represented by one H_2O molecule interacting in the initial geometry with H^*_{ads}; and (C) Bader charges[3] in |e| on H^*_{ads} in the gas phase and with explicit solvation.

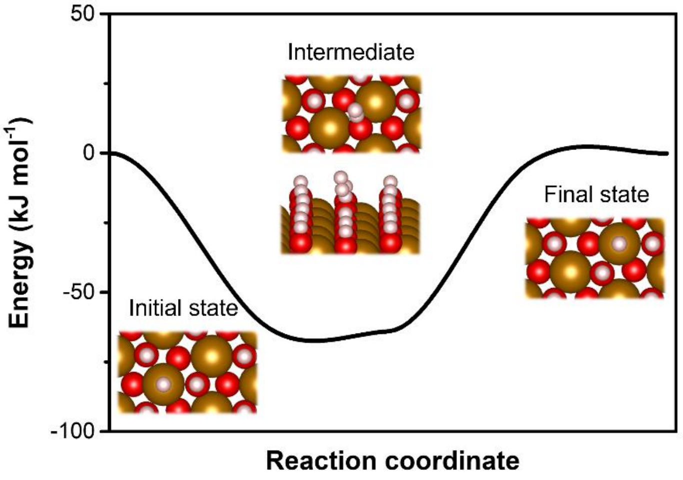

Figure S9. Reaction profile of H^*_{ads} migration between two top Fe sites on the $Fe(OH)_2(001)$ surface.

Table S8. Imaginary vibrational modes and their frequencies for transition states of Tafel recombination reactions.

Surface	Imaginary vibrational mode	Corresponding frequency (cm^{-1})
Fe(110)	No TS found	
$S_{1/8\ ML}$-Fe(110)	No TS found	
$S_{1/4\ ML}$-Fe(110)	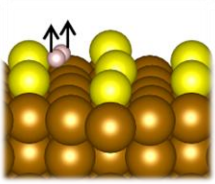	–228
$S_{1/2\ ML}$-Fe(110)	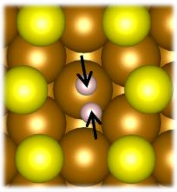	–749
$FeS_m(001)$	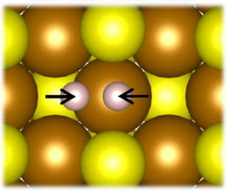	–446
$O_{1/4\ ML}$-Fe(110)	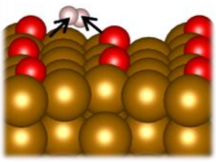	(–59)*
$OH_{1/4\ ML}$-Fe(110)	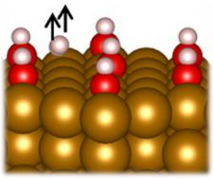	–314

* While both the CI-NEB and dimer methods identified a TS, the frequency calculation struggled to identify a strong imaginary vibrational mode along the reaction pathway due to its flat energy profile (see Figure 6A).

References

(1) Blöchl, P. E.; Jepsen, O.; Andersen, O. K. Improved Tetrahedron Method for Brillouin-Zone Integrations. *Phys. Rev. B* **1994**, *49* (23), 16223–16233. https://doi.org/10.1103/PhysRevB.49.16223.

(2) Wang, V.; Xu, N.; Liu, J.-C.; Tang, G.; Geng, W.-T. VASPKIT: A User-Friendly Interface Facilitating High-Throughput Computing and Analysis Using VASP Code. *Comput. Phys. Commun.* **2021**, *267*, 108033. https://doi.org/10.1016/j.cpc.2021.108033.

(3) Tang, W.; Sanville, E.; Henkelman, G. A Grid-Based Bader Analysis Algorithm without Lattice Bias. *J. Phys. Condens. Matter* **2009**, *21* (8), 084204. https://doi.org/10.1088/0953-8984/21/8/084204.
